# TD-DFT insight into photodissociation of the Co-C bond in coenzyme B_12_

**DOI:** 10.3389/fchem.2013.00041

**Published:** 2014-02-05

**Authors:** Hui Liu, Karina Kornobis, Piotr Lodowski, Maria Jaworska, Pawel M. Kozlowski

**Affiliations:** ^1^Department of Chemistry, University of LouisvilleLouisville, KY, USA; ^2^Department of Theoretical Chemistry, Institute of Chemistry, University of SilesiaKatowice, Poland

**Keywords:** coenzyme B_12_, Co-C bond, photodissociation, ribosylcobalamin, time-dependent density functional theory

## Abstract

Coenzyme B_12_ (AdoCbl) is one of the most biologically active forms of vitamin B_12_, and continues to be a topic of active research interest. The mechanism of Co-C bond cleavage in AdoCbl, and the corresponding enzymatic reactions are however, not well understood at the molecular level. In this work, time-dependent density functional theory (TD-DFT) has been applied to investigate the photodissociation of coenzyme B_12_. To reduce computational cost, while retaining the major spectroscopic features of AdoCbl, a truncated model based on ribosylcobalamin (RibCbl) was used to simulate Co-C photodissociation. Equilibrium geometries of RibCbl were obtained by optimization at the DFT/BP86/TZVP level of theory, and low-lying excited states were calculated by TD-DFT using the same functional and basis set. The calculated singlet states, and absorption spectra were simulated in both the gas phase, and water, using the polarizable continuum model (PCM). Both spectra were in reasonable agreement with experimental data, and potential energy curves based on vertical excitations were plotted to explore the nature of Co-C bond dissociation. It was found that a repulsive ^3^(σ_Co−C_ → σ^*^_Co−C_) triplet state became dissociative at large Co-C bond distance, similar to a previous observation for methylcobalamin (MeCbl). Furthermore, potential energy surfaces (PESs) obtained as a function of both Co-C_Rib_ and Co-N_Im_ distances, identify the S_1_ state as a key intermediate generated during photoexcitation of RibCbl, attributed to a mixture of a metal-to-ligand charge transfer (MLCT) and a σ bonding-ligand charge transfer (SBLCT) states.

## Introduction

**Figure 1:**
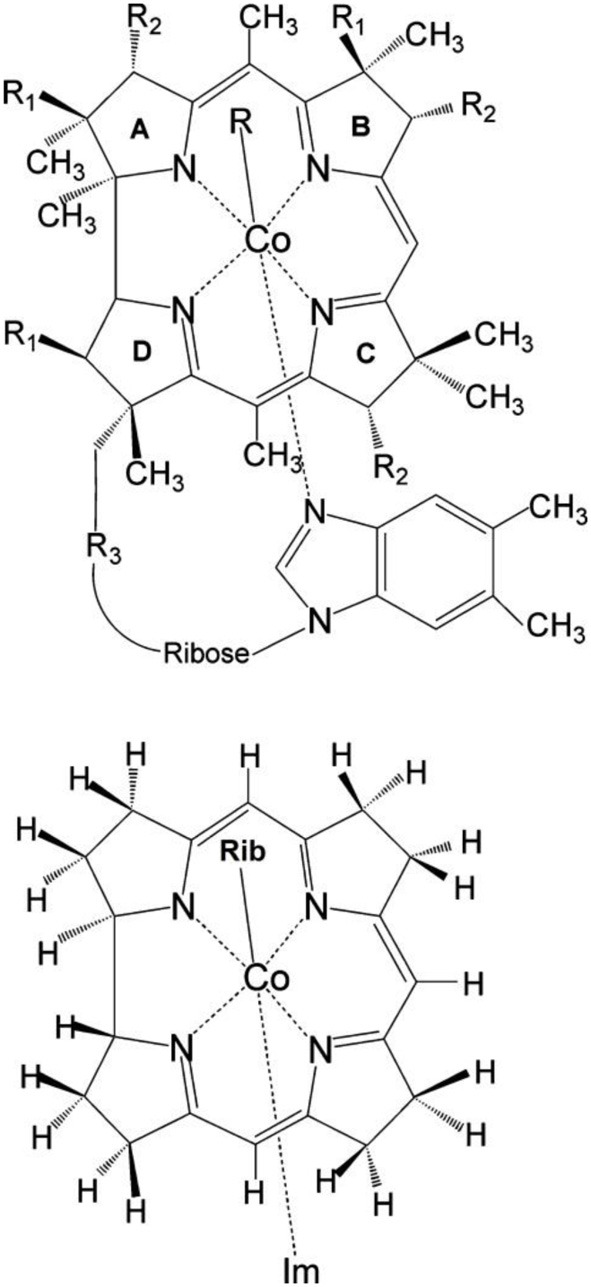
**Upper:** Molecular structure of vitamin B_12_ derivatives where R = Me, Ado, Et, Prop, CN, OH or N_3_ where R_1_ =CH_2_CONH_2_, R_2_ = CH_2_CH_2_CONH_2_, and R_3_ = (CH_2_)_2_CONHCH_2_CH(CH_3_)OPO^−^_3_. **Lower:** Structural model of RibCbl employed in present work (Rib refers to ribosyl with 5-hydroxyl group substituted by H atom). Reprinted (adapted) with permission from Jaworska et al. (2007). Copyright (2014) American Chemical Society.

Vitamin B_12_ derivatives (Figure 1) are a group of organometallic complexes that act as biologically active cofactors in many enzymatic reactions (Dolphin et al., 1982; Banerjee, 1997, 1999, 2001, 2003; Ludwig and Matthews, 1997; Kräutler et al., 1998; Marzilli, 1999; Toraya, 2000; Matthews, 2001; Banerjee and Ragsdale, 2003; Toraya, 2003; Brown, 2005; Randaccio et al., 2006, 2007). In addition to their pivotal roles in a variety of enzymatic processes, the B_12_ derivatives possess complex photophysical and photochemical properties (Endicott and Ferraudi, 1977; Endicott and Netzel, 1979; Rao and Symons, 1982; Chen and Chance, 1990, 1993; Sakaguchi et al., 1990; Chagovetz and Grissom, 1993; Grissom and Chagovetz, 1993; Lott et al., 1995; Natarajan and Grissom, 1996; Kruppa et al., 1997; Walker et al., 1998a,b; Shiang et al., 1999, 2006; Yoder et al., 2001; Cole et al., 2002; Sension et al., 2004, 2005a,b; Harris et al., 2007). The relatively weak organometallic Co-R bond (R = alkyl, hydroxyl, water, cyanide) in these compounds can undergo photodissociation under conditions of simple photon excitation, depending on the nature of the upper axial ligand. Cobalamin complexes with the alkyl axial ligands, such as enzymatically competent methylcobalamin (MeCbl) and adenosylcobalamin (AdoCbl), and their analogs, ethylcobalamin (EtCbl) and propylcobalamin (PropCbl), undergo photodissociation upon exposure to light (Walker et al., 1998a,b; Shiang et al., 1999; Yoder et al., 2001; Cole et al., 2002; Sension et al., 2004, 2005a,b; Harris et al., 2007). In certain cases, the nature of events following photon excitation is additionally influenced by a wavelength of exciting light, as found for MeCbl (Harris et al., 2007). On the other hand, the Co-R bond in cyannocobalimin (CNCbl) and non-alkylcobalamin analogs, such as azidocobalamin (N_3_Cbl) and aquocobalamin (H_2_OCbl^+^), are rather photostable (Shiang et al., 2006).

The photochemistry of cobalt corrinoids has been probed using various experimental techniques such as laser flash photolysis (Endicott and Ferraudi, 1977; Endicott and Netzel, 1979; Chen and Chance, 1990; Chagovetz and Grissom, 1993; Lott et al., 1995), continuous wave (CW) photolysis (Chen and Chance, 1993), kinetic magnetic field effect (MFE) (Grissom and Chagovetz, 1993; Natarajan and Grissom, 1996), chemically induced dynamic electron polarization (CIDEP) (Rao and Symons, 1982; Sakaguchi et al., 1990) as well as nuclear polarization (CIDNP) (Kruppa et al., 1997). Systematic studies of photolysis in B_12_ alkyl-derivatives (MeCbl, EtCbl, PropCbl, and AdoCbl) using pump-probe transient absorption spectroscopy found that photoproduct yields for alkylcobalamins are different when excited at 400, or 520/530 nm (Walker et al., 1998a,b; Shiang et al., 1999; Yoder et al., 2001; Cole et al., 2002; Sension et al., 2004, 2005a,b; Harris et al., 2007). MeCbl for example, produces a mixture of 27% cob(II)alamin and 73% cob(III)alamin when excited at 400 nm as a result of partitioning between Co-C homolysis and heterolytic cleavage. Heterolytic bond cleavage products were identified as a metal-to-ligand charge transfer (MLCT) state upon excitation at 400 nm (Cole et al., 2002), and only metastable cob(III)alamin for 520 nm (Shiang et al., 1999). In contrast, photoexcitation of AdoCbl produces wavelength independent photoproducts at 400 and 520 nm, that correspond to an intermediate state observed as decay over a 14 ps time scale, of two relaxed radical pairs. These radical pairs were characterized as cob(II)alamin and an adenosyl-based radical. This observation corresponds to Co-C homolysis, or a trapped excited state with a weak Co-C bond (Shiang et al., 1999). Furthermore, although the photoproducts of AdoCbl were found to be insensitive to excitation wavelength, environmental effects have been shown to influence its photophysics (Yoder et al., 2001).

The photodissociation of Co-C in cobalamins has also been investigated computationally by means of time-dependent density functional theory (TD-DFT) (Jaworska et al., 2007; Lodowski et al., 2009, 2011; Kumar and Kozlowski, 2012). In the case of MeCbl, the key metastable photoproduct present during the photolysis process was identified as an S_1_ state with MLCT character (Jaworska et al., 2007), in accordance with experiment. Further analysis of the electron density map of S_1_ and S_0_ states indicated an additional contribution from σ bond, revealing that σ bonding-ligand charge transfer (SBLCT) character was mixed with MLCT (Lodowski et al., 2009). To explain the nature of the Co-C photo-scission in MeCbl, the presence of a repulsive ^3^(σ_Co−C_ → σ^*^_Co−C_) triplet state was proposed based on TD-DFT analysis of electronically excited states along the stretched Co-C bond. The same approach has also been applied to EtCbl, and the presence of a repulsive triplet state was used to explain the energetic differences between MeCbl and EtCbl, as well as why the photolysis mechanism is wavelength dependent in the case of MeCbl (Lodowski et al., 2009).

A similar study was performed for vitamin B_12_ (CNCbl) to explain its photostability and the nature of its low-lying excited states (Lodowski et al., 2011). Potential energy curves for low-lying excited states along the Co-CN coordinate reveal that CNCbl has a repulsive triplet state, but is not dissociative. It was found that the CNCbl potential energy surface (PES) of the S_1_ state, when represented as a function of axial distances, had two energy minima. The first minima was located above the S_0_ minimum as an excitation with mixed π → π^*^/MLCT/SBLCT character, similar to the alkylcobalamin S_1_ state. The second minimum was found at longer Co-N_Im_ and Co-CN bond lengths, and was characterized as ligand-to-metal charge-transfer (LMCT) excitation.

Interestingly, the Co-OH bond in hydroxycobalamin (HOCbl) is dissociative upon light exposure above 300 nm (Shell and Lawrence, 2011), although it is among the non-alkylcobalamins. Photolysis of HOCbl has also been studied by TD-DFT, by focusing on the Co-OH cleavage (Kumar and Kozlowski, 2012). TD-DFT results suggest that the photoactivity of HOCbl is mediated by the repulsive ^1^(n+d_Co_→ σ^*^_Co−OH_) singlet state whose energy drops with Co-OH bond stretching, to yield the cob(II)alamin and hydroxyl radical.

Although spectroscopic techniques have been extensively applied to alkyl- and non-alkylcobalamins, the mechanism of Co-C bond dissociation is still not well understood. The aim of this study is to provide the further insight into the photodissociation mechanism of the Co-C bond in the coenzyme B_12_ (AdoCbl, Figure 1) by TD-DFT computations. To accomplish this we employed the simplified structural model of AdoCbl, (RibCbl, Figure 1), in which the Ado group was simplified to a ribosyl (Rib) moiety. The intermediates involved in the photodissociation process were identified by systematically analyzing the nature of low-lying excited states as well as their changes along stretched Co-C_Rib_ coordinate. Finally, in order to provide a more accurate picture of key intermediates, the PESs of ground state and low-lying singlet states were analyzed as a function of both Co-C_Rib_ and Co-N_Im_ axial bond distances.

## Computational details

All calculations reported in this work were based on nonlocal DFT with the gradient-corrected Becke-Perdew (BP86) (Perdew, 1986; Becke, 1993) functional and the TZVP basis set, as implemented in the Gaussian 09 (Frisch et al., 2009) or TURBOMOLE (Ahlrichs et al., 1989; Treutler and Ahlrichs, 1995; Furche and Ahlrichs, 2002; Furche and Rappoport, 2005; TURBOMOLE^1^) suites of programs for electronic structure calculations. The BP86 functional has been previously selected as the most appropriate for predicting both the structural and electronic properties of B_12_ cofactors, including Co-C bond dissociation energy (BDE) (Jensen and Ryde, 2003; Rovira et al., 2004; Kuta et al., 2006; Kozlowski et al., 2007; Rovira and Kozlowski, 2007; Galezowski et al., 2008; Lodowski et al., 2009, 2011; Kornobis et al., 2011, 2013). To account for environmental effects on geometries and electronic properties, ground states of RibCbl model (Im-[Co^III^(corrin)]-Rib^+^, see Figure S1) were computed in the gas phase as well as in water via the Polarizable Continuum Model (PCM) (Miertuš et al., 1981; Cammi and Tomasi, 1995) as implemented in Gaussian 09.

Vertical excitations were obtained in both the gas phase and water solvent. Low-lying excited states were calculated from their corrdepsonding ground state geometries by TD-DFT, and both singlet and triplet excitations have been calculated to forty states (Tables S1–S4). To simulate the photodissociation process of RibCbl, the Co-C_Rib_ bond was stretched with a step size of 0.05 Å and the DFT/BP86/TZVP optimization was repeated at each point, followed by TD-DFT calculations at the BP86/TZVP level of theory. In addition, PESs of the ground state and key singlet states were plotted by stretching axial bonds in step of 0.05 Å. At each grid point, the geometry was optimized by DFT/BP86/TZVP, and the vertical excitation was obtained for S_1_ and S_2_ states.

## Results and discussion

### Structural model of RibCbl

The initial molecular structure of RibCbl was obtained from the high-resolution crystal structure of AdoCbl (Ouyang et al., 2004). The full structure of AdoCbl (Figure 1) consists of Co^III^ equatorially cooridinated via four nitrogen atoms to the corrin ring. The lower axial ligand, dimethylbenzimidazole (DBI) coordinates to the central Co atom via N_ax_, with the other N atom bound to the side of the corrin plane by a nucleotide loop. In the actual AdoCbl structure, the upper Ado ligand is 5′-deoxy-5′-adenosine.

To reduce computational cost, calculations were performed using the simplified structural model of RibCbl, denoted Im-[Co^III^(corrin)]-Rib^+^for consistency with previous studies (Jaworska et al., 2007; Lodowski et al., 2009, 2011). In the RibCbl model (see Figure 1), the Ado group was simplified to ribosyl (Rib) by omitting the purine ring, leaving only ribose in the upper corrin plane. The corrin ring was truncated by replacing the side chains with hydrogen atoms, and DBI with imidazole (Im). The resulting overall charge of the system was +1 due to removal of the PO^−^_4_ nucleotide loop from below the plane. Previous studies have demonstrated that such simplified structural models accurately reproduce the electronic and spectroscopic properties of the full B_12_ structure (Jaworska and Lodowski, 2003; Jensen and Ryde, 2003, 2009; Stich et al., 2003, 2004; Jaworska et al., 2005, 2007; Kuta et al., 2006, 2009; Liptak and Brunold, 2006; Kozlowski et al., 2007, 2012; Rovira and Kozlowski, 2007; Galezowski et al., 2008; Lodowski et al., 2009, 2011). More inclusive structural models of coenzyme B_12_ will be employed in future studies utilizing multiple levels of theory.

Optimized RibCbl structures correspond to equilibrium geometries where Co-C_Rib_ = 2.02 Å and Co-N_Im_ = 2.21 Å in the gas phase, and Co-C_Rib_ = 2.02 Å and Co-N_Im_ = 2.19 Å in water (PCM). Optimized axial bond lengths are in reasonable agreement with X-ray data of Randaccio et al. (Ouyang et al., 2004) with corresponding experimental bond lengths of 2.04 Å for Co-C_Ado_ and 2.23 Å for Co-N_DBI_, respectively. Molecular orbital (MO) energies and fragment contributions of RibCbl are collected in Table S5 for the gas phase, and Table S6 for water (PCM). The relevant MO diagram can be found in supporting information as Figure S2.

Frontier orbitals of RibCbl were determined by fragment analysis to be mainly corrin π, π^*^, and metal d orbitals. In particular, HOMO, HOMO−2, −4, −6, and LUMO+1, +2, +3 show major contributions from d_Co_ and π^*^ while HOMO−3, −5, −8, −9, −10, and LUMO+3 have increased weights from the Rib group. On the other hand, LUMO and LUMO+4 are composed of nearly pure π orbitals of the corrin ring. Finally, HOMO-7 and LUMO+5 are both composed of excitations from the imidazole group. The change of environment from gas phase to water solution (Table S6) did not influence the character of the MOs significantly, differences being attributed to reversion of energies between the HOMO-5 and −7 in the gas, and HOMO-3 and −4 in the PCM model.

### Electronically excited states of RibCbl

**Figure 2:**
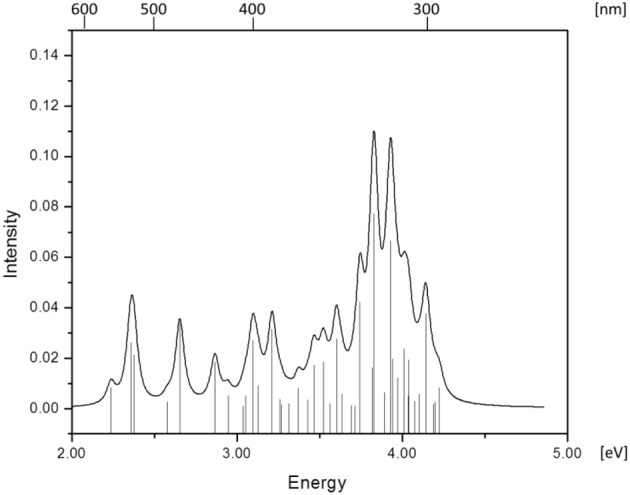
**Absorption spectrum of RibCbl calculated in gas phase with BP86/TZVP**.

**Figure 3:**
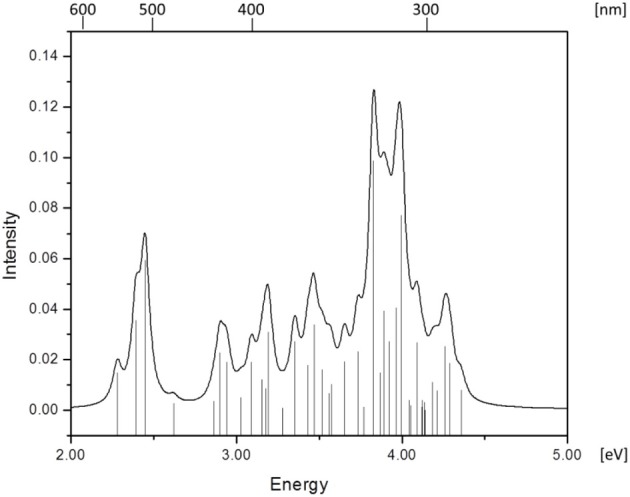
**Absorption spectrum of RibCbl calculated in water solution with BP86/TZVP**.

Simulated absorption (Abs) spectra generated from TD-DFT/BP86/TVZP vertical excitation energies with transition dipole moments, using corresponding optimized RibCbl geometries in the gas phase and in water, are shown in Figures 2, 3 respectively. Since hybrid B3LYP functional underestimates BDE as well as produce low-lying states as pure π_corrin_ → π^*^_corrin_ excitations, we employed BP86 throughout this study which has been proved to produce correct BDE for both MeCbl and AdoCbl (Kuta et al., 2006; Kozlowski et al., 2007, 2012; Galezowski et al., 2008; Kornobis et al., 2011, 2013; Kumar et al., 2013). BP86 functional was also frequently applied in QM/MM calculations for the enzyme-active cofactors (Kumar et al., 2012; Kumar and Kozlowski, 2013). In both the gas phase and solvent-based calculations, forty excited states were calculated to cover a range appropriate for electronic states potentially involved in coenzyme B_12_ photolysis. The lowest ten singlet states are listed in Table 1, and the lowest ten triplet states are listed in Table 2 for the gas phase. In addition, the corresponding solvent-based calculations are listed in Table 3 for singlet states and Table 4 for triplet states. Complete lists of the forty excited states for each system can be found in the supporting information (Tables S1–S4). Taking into account that light exposure used in experimental studies was no shorter than 400 nm to induce Co-C cleavage (Walker et al., 1998a,b; Shiang et al., 1999; Yoder et al., 2001; Cole et al., 2002; Sension et al., 2004, 2005a,b; Harris et al., 2007), in further discussion we will focus on the low-energy region.

**Table 1:** **The lowest ten singlet states for RibCbl received from TDDFT/TZVP gas phase calculations**.

	**E (eV)**	**λ (nm)**	***f***	**Coeff**.			**Character**
S_1_	2.24	554.1	0.0082	74	143 → 145	H-1 → L	π + d_xz_/d_z^2^_ → π*
				18	142 → 145	H-2 → L	d_xz_ + π → π*
S_2_	2.36	526.1	0.0261	74	144 → 145	H → L	d_yz_ + π → π*
				14	142 → 145	H-2 → L	d_xz_ + π → π*
S_3_	2.37	522.1	0.0213	58	142 → 145	H-2 → L	d_xz_ + π → π*
				12	144 → 145	H → L	d_yz_ + π → π*
				12	141 → 145	H-3 → L	n_Rib_/σ_Rib_ + d_x^2^−y^2^_ +π → π*
				12	143 → 145	H-1 → L	π + d_xz_/d_z^2^_ → π*
S_4_	2.58	481.1	0.0026	61	140 → 145	H-4 → L	d_x^2^_−y^2^ → π*
				35	141 → 145	H-3 → L	n_Rib_/σ_Rib_ + d_x^2^_−y^2^ + π → π*
S_5_	2.65	467.2	0.0337	44	141 → 145	H-3 → L	n_Rib_/σ_Rib_ + d_x^2^_−y^2^ + π →π*
				33	140 → 145	H-4 → L	d_x^2^_−y^2^ →π*
				10	143 → 145	H-1 → L	π + d_xz_/d_z^2^_ →π*
S_6_	2.87	432.5	0.0186	65	144 → 146	H → L+1	d_yz_ + π → d_xy_ −n + π*
				12	143 → 146	H-1 → L+1	π + d_xz_/d_z^2^_ → d_xy_ −n + π*
S_7_	2.95	421.0	0.0051	62	143 → 146	H-1 → L+1	π + d_xz_/d_z^2^_ → d_xy_ −n + π*
				13	144 → 146	H → L+1	d_yz_ + π → d_xy_ −n + π*
				9	144 → 148	H → L+3	d_yz_ + π → σ*(d_z^2^_) + n
S_8_	3.04	408.2	0.0008	36	144 → 148	H → L+3	d_yz_ + π → s*(d_z^2^_) + n
				27	144 → 147	H → L+2	d_yz_ + π → d_xy_ −n + π*
				18	139 → 145	H-5 → L	n_Rib_/σ_Rib_ + d_xz_/d_z^2^_ + π →π*
S_9_	3.05	406.1	0.0050	41	139 → 145	H-5 → L	n_Rib_/σ_Rib_ + d_xz_/d_z^2^_ + π → π*
				31	144 → 148	H → L+3	d_yz_ + π → s*(d_z^2^_) + n
				10	142 → 146	H-2 → L+1	d_xz_ + π → d_xy_ −n + π*
S_10_	3.10	400.5	0.0269	43	144 → 147	H → L+2	d_yz_ + π → d_xy_ −n + π*
				28	139 → 145	H-5 → L	n_Rib_/σ_Rib_ + d_xz_/d_z^2^_ + π → π*

**Table 2:** **The lowest ten triplet states for RibCbl received from TDDFT/TZVP gas phase calculations**.

	**E (eV)**	**λ (nm)**	**Coeff**.			**Character**
T_1_	1.72	720.0	49	144 → 145	H → L	d_yz_ + π →π*
T_2_	1.94	639.9	48	143 → 145	H-1 → L	π + d_xz_/d_z^2^_ →π*
T_3_	2.15	577.6	47	142 → 145	H-2 → L	d_xz_ + π →π*
T_4_	2.25	551.8	35	144 → 146	H → L+1	d_yz_ + π → d_xy_ −n + π*
			10	144 → 147	H → L+2	d_yz_ + π → d_xy_ −n + π*
T_5_	2.39	518.7	30	142 → 146	H-2 → L+1	d_xz_ + π → d_xy_ −n + π*
			8	142 → 147	H-2 → L+2	d_xz_ + π → d_xy_ −n + π*
T_6_	2.47	501.4	28	141 → 145	H-3 → L	n_Rib_/σ_Rib_ + d_x^2^−y^2^_ + π→π*
			18	140 → 145	H-4 → L	d_*x*^2^−*y*^2^_ → π*
T_7_	2.48	499.6	26	140 → 145	H-4 → L	d_*x*^2^−*y*^2^_ →π*
			18	141 → 145	H-3 → L	n_Rib_/σ_Rib_ + d_*x*^2^−*y*^2^_ + π→π*
T_8_	2.52	491.2	29	140 → 146	H-4 → L+1	d_*x*^2^−*y*^2^_ → d_*xy*_ −n + π*
			12	140 → 147	H-4 → L+2	d_*x*^2^−*y*^2^_→ d_*xy*_ −n + π*
			5	141 → 146	H-3 → L+1	n_Rib_/σ_Rib_ + d_*x*^2^−*y*^2^_ + π→ d_*xy*_ −n + π*
T_9_	2.55	487.0	15	144 → 148	H → L+3	d_*yz*_ + π→ σ*(d_*z*^2^_) + n
			9	144 → 147	H → L+2	d_*yz*_ + π→ d_*xy*_ −n + π*
			8	143 → 146	H-1 → L+1	π + d_*xz*_/d_*z*^2^_ → d_*xy*_ −n + π*
			5	144 → 146	H → L+1	d_*yz*_ + π→ d_*xy*_ −n + π*
T_10_	2.58	479.8	11	143 → 148	H-1 → L+3	π + d_*xz*_/d_*z*^2^_ → σ*(d_*z*^2^_)+ n
			10	143 → 146	H-1 → L+1	π + d_*xz*_/d_*z*^2^_ → d_*xy*_ −n + π*
			6	144 → 148	H → L+3	d_*yz*_ + π→ σ*(d_*z*^2^_) + n
			6	143 → 147	H-1 → L+2	π + d_*xz*_/d_*z*^2^_ → d_*xy*_ −n + π*
			4	144 → 147	H → L+2	d_*yz*_ + π→ d_*xy*_ −n + π*
			4	142 → 147	H-2 → L+2	d_*xz*_ + π→ d_*xy*_ −n + π*

**Table 3:** **The lowest ten singlet states for RibCbl received from TDDFT/TZVP PCM (water) calculations**.

	**E(eV)**	**λ (nm)**	***f***	**Coeff**.			**Character**
S_1_	2.28	543.4	0.0150	27	142 → 145	H-2 → L	d_xz_ + π → π*
				56	143 → 145	H-1 → L	π + d_yz_/d_z^2^_ → π*
				15	144 → 145	H → L	d_yz_ + π → π*
S_2_	2.39	518.9	0.0353	74	144 → 145	H → L	d_yz_ + π → π*
S_3_	2.45	506.4	0.0597	61	142 → 145	H-2 → L	d_xz_ + π → π*
				29	143 → 145	H-1 → L	π + d_yz_/d_z^2^_ → π*
S_4_	2.62	473.5	0.0024	97	141 → 145	H-3 → L	d_*x*^2^−*y*^2^_ → π*
S_5_	2.86	434.0	0.0043	62	144 → 146	H → L+1	d_yz_ + π → d_xy_ −n + π*
				16	140 → 145	H-4 → L	n_Rib_/σ_Rib_ → π*
S_6_	2.90	427.7	0.0235	62	140 → 145	H-4 → L	n_Rib_/σ_Rib_ → π*
				23	143 → 146	H-1 → L+1	π + d_yz_/d_z^2^_ → d_xy_ −n + π*
S_7_	2.94	421.8	0.0186	53	143 → 146	H-1 → L+1	π + d_yz_/d_z^2^_ → d_xy_ −n + π*
				14	140 → 145	H-4 → L	n_Rib_/σ_Rib_ → π*
				14	144 → 146	H → L+1	d_yz_ + π → d_xy_ −n + π*
S_8_	3.02	410.0	0.0053	59	144 → 148	H → L+3	d_yz_ + π → σ*(d_z^2^_) + n
				20	144 → 147	H → L+2	d_yz_ + π → d_xy_ −n + π*
S_9_	3.09	401.4	0.0185	55	144 → 147	H → L+2	d_yz_ + π → d_xy_ −n + π*
				19	144 → 148	H → L+3	d_yz_ + π → σ*(d_z^2^_) + n
S_10_	3.16	392.7	0.0112	35	139 → 145	H-5 → L	π_Im_ → π*
				33	143 → 148	H-1 → L+3	π + d_yz_/d_z^2^_ → σ*(d_z^2^_) + n
				15	143 → 147	H-1 → L+2	π + d_yz_/d_z^2^_ → d_xy_ −n + π*
				9	138 → 145	H-6 → L	π + d_yz_ → π*

**Table 4:** **The lowest ten triplet states for RibCbl received from TDDFT/TZVP PCM (water) calculations**.

	**E (eV)**	**λ (nm)**	**Coeff**.			**Character**
T_1_	1.75	708.3	48	144 → 145	H → L	d_yz_ + π → π*
T_2_	1.94	637.8	47	143 → 145	H-1 → L	π + d_yz_/d_*z*^2^_ → π*
T_3_	2.19	567.1	45	142 → 145	H-2 → L	d_xz_ + π → π*
T_4_	2.26	548.4	33	144 → 146	H → L+1	d_yz_ + π → d_xy_ −n + π*
			7	144 → 147	H → L+2	d_yz_ + π → d_xy_ −n + π*
T_5_	2.40	516.3	34	142 → 146	H-2 → L+1	d_xz_ + π → d_xy_ −n + π*
			6	142 → 147	H-2 → L+2	d_xz_ + π → d_xy_ −n + π*
T_6_	2.50	496.6	27	141 → 145	H-3 → L	d_*x*^2^−*y*^2^_ → π*
			15	141 → 146	H-3 → L+1	d_*x*^2^−*y*^2^_ → d_xy_ −n + π*
			5	141 → 147	H-3 → L+2	d_*x*^2^−*y*^2^_ → d_xy_ −n + π*
T_7_	2.52	492.7	12	144 → 147	H → L+2	d_yz_ + π → d_xy_ −n + π*
			17	144 → 148	H → L+3	d_yz_ + π → σ*(d_*z*^2^_) + n
			5	141 → 145	H-3 → L	d_*x*^2^−*y*^2^_ → π*
			4	141 → 146	H-3 → L+1	d_*x*^2^−*y*^2^_ → d_xy_ −n + π*
T_8_	2.54	488.3	16	141 → 145	H-3 → L	d_*x*^2^−*y*^2^_ → π*
			15	141 → 146	H-3 → L+1	d_*x*^2^−*y*^2^_ → d_xy_ −n + π*
			5	141 → 147	H-3 → L+2	d_*x*^2^−*y*^2^_ → d_xy_ −n + π*
			4	144 → 147	H → L+2	d_yz_ + π → d_xy_ −n + π*
T_9_	2.61	475.6	15	143 → 146	H-1 → L+1	π + d_yz_/d_*z*^2^_ → d_xy_ −n + π*
			10	143 → 147	H-1 → L+2	π + d_yz_/d_*z*^2^_ → d_xy_ −n + π*
			9	143 → 148	H-1 → L+3	π + d_yz_/d_*z*^2^_ → σ*(d_*z*^2^_) + n
			6	142 → 147	H-2 → L+2	d_xz_ + π → d_xy_ −n + π*
T_10_	2.71	457.6	21	142 → 148	H-2 → L+3	d_xz_ + π → σ*(d_*z*^2^_) + n
			13	142 → 147	H-2 → L+2	d_xz_ + π → d_xy_ −n + π*
			7	143 → 148	H-1 → L+3	π + d_yz_/d_*z*^2^_ → σ*(d_*z*^2^_) + n

Since the studied RibCbl model is a truncated form of full coenzyme B_12_, no direct comparison to the experimental spectrum can be made. However, the predicted spectra can be correlated to that of AdoCbl with good agreement (Shiang et al., 1999). Several calculated electronic transitions can be assigned to experimental spectral bands. Specifically, the S_2_ at 2.36 eV and the S_3_ at 2.37 eV agree well with the experimental transition at 2.35 eV (528 nm), the S_6_ at 2.87 eV agrees with the experimental transition at 2.90 eV (428 nm), and the S_10_ state at 3.10 eV can be assigned to absorption observed at 3.07 eV (404 nm). The same trend applies to PCM geometries (Table 3). Namely, the S_2_, S_3_, S_6_, and S_9_ can all be correlated with experimental absorption bands for AdoCbl observed at 2.35 eV (528 nm), 2.90 eV (428 nm), and 3.07 eV (404 nm), respectively. Furthermore, the simulated RibCbl Abs spectra are also in reasonable agreement with the simulated AdoCbl spectra published by Andruniow et al. (2009) further supporting the ability of the truncated AdoCbl model, (i.e., RibCbl) to accurately reproduce the experimental spectroscopic features of AdoCbl.

The absorption spectrum of RibCbl calculated in the gas phase at the BP86/TZVP level is shown in Figure 2, and assignments for the lowest forty singlet states are collected in Table S1. Based on these results it can be deduced that the α/β bands are located in the low energy region from 2.00 to 2.80 eV, covering first five calculated singlet transitions, the D/E band ranges from 2.80 to 3.30 eV and is composed of multiple excitations, and the high-energy region, or so-called γ band, has significant intensity covering several transitions from 3.30 to 4.40 eV. Based on the nature of the calculated electronic excited states (Table 1), the α/β bands involve primarily d_Co_/π → π^*^ contributions, the D/E bands result from various electronic transitions with more contributions from the Rib group, and the same tendency is observed in the γ band, where oscillator strength increases significantly. It should be noted that ribose increases its role in photoelectronic transitions in the high energy region, i.e., the D/E and γ bands, respectively.

Table 1 summarizes the ten lowest calculated excited singlet states of RibCbl in the gas phase. As shown in Table 1, the S_1_ excitation present at 2.24 eV, carries only a small oscillator strength (0.008). The major contribution from the S_1_ state is an excitation from HOMO-1 to LUMO, in which π + d_xz_/d_z^2^_ → π^*^ is dominant. The MO diagram (Figure S2), shows that S_1_ excitation also involves a transition from a σ orbital of axial bonding to the π^*^ of the corrin ring, and is identified as SBLCT (Lodowski et al., 2009). The second noticeable contribution from the S_1_ state is the transition from HOMO-2 to LUMO, identified as π + d_xz_ → π^*^. Based on the MO diagram it is apparent that LUMO is a pure corrin π^*^ orbital, to which all the excitations in the α/β band generally take place. The next two states, the S_2_ and S_3_, are adjacent closely in energy. The most significant components of the S_2_ and S_3_ states are HOMO → LUMO and HOMO-2 → LUMO, where HOMO is a mixture of π and d_yz_ and HOMO-2 refers to π and d_xz_. Therefore, the d to π^*^ transition is dominant in this region, and should be described as MLCT transition (Cole et al., 2002; Jaworska et al., 2007; Lodowski et al., 2009, 2011).

The S_4_ state has even less oscillator strength intensity compared to that of S_1_, in which both HOMO-3 and HOMO-4 contribute almost evenly to excitation at 2.58 eV. HOMO-3 derives from ribose, according to MO diagram, while HOMO-4 is composed from the Co d_x^2^ −y^2^_ orbital. Coincidently, the S_5_ involves the same set of orbitals which contribute to the S_4_ state. The only difference, considering their specific characters, is that the S_5_ state mixes a minor contribution from the HOMO-1→ LUMO transition. However, the larger oscillator strength of the S_5_ state makes it the strongest component of the α/β band.

Electronic transitions in the D/E band are more complex than those in the α/β band, and the oscillator strengths are noticeably smaller. This region can be represented by electronic transitions from the S_6_ to S_14_ with three strong excitations occurring at 2.87 (S_6_), 3.10 (S_10_), and 3.21 eV (S_12_) (Figure 2). The S_6_ and S_10_ have d_Co_/π → π^*^ character while the S_12_ shows a d_Co_/π → σ^*^_Co−C_ nature. The other small intensity transitions in this region are of similar character.

More than twenty singlet states can be associated with γ band ranging from 3.31 (S_15_) to 4.23 eV (S_40_) (Table S1). Among them, the S_25_ and S_27_ have the largest transition dipole moment and are composed of n_Rib_/σ_Rib_ + d_xz_/d_z^2^_ + π →d_xy_ −n + π^*^ transitions with a minor d_Co_/ π → π^*^ contribution. The S_29_ is dominated by d_Co_/π → π^*^ transitions, while the S_32_ has d_Co_/π → π^*^_Im_ character. Near the end of the simulated spectrum, another noticeable transition of n_Rib_/σ_Rib_ + d_xz_/d_z^2^_ + π → σ^*^(d_z^2^_) + n type is found at 4.14 eV corresponding to the S_37_ state (Table S1).

Since triplet excitations have zero transition dipole moments, we will not take them into account for spectral analysis. The first three triplet states are energetically below the S_1_ (2.24 eV), where the T_1_ occurs at 1.72 eV from an electronic transition of HOMO→LUMO, while T_2_ and T_3_ are transitions at 1.94 and 2.15 eV, originating from HOMO-1→LUMO and HOMO-2→LUMO, respectively. All three electronic transitions can be described as d_Co_/π → π^*^ type (see Table 2 and Table S2).

The Abs spectrum of RibCbl calculated in water solution using the same level of theory was plotted in Figure 3. The energy of the γ band remains unchanged in comparison to gas phase calculation. However, the α/β band is narrowed due to the blue shift of the S_5_ to the D/E region. The S_1_, S_2_, and S_3_ are assigned to d_Co_/π → π^*^ transition. A significant contribution to S_1_ state is composed of a π + d_xz_/d_z^2^_ → π^*^ excitation and, like in the gas phase, is characterized as SBLCT. The S_4_ has pure d_x^2^ −y^2^_ → π^*^ character. The D/E band covers states S_5_–S_13_. The states S_5_ and S_7_ are identified as d/π → π^*^/d−n excitations with a small admixture of long range CT (LRCT) type transitions (n_Rib_/σ_Rib_ → π^*^) (Andruniow et al., 2009). The S_6_ state at 2.90 eV is a mixture of a dominant LRCT, and minor d/π → π^*^/d−n character. In contrast, the S_7_ state is mainly composed of a d_Co_/π → π^*^ transition, with minor contributions from SBLCT. The other intense singlet transitions in this region are assigned to the d_Co_/π → π^*^ transition, which is consistent with singlet excited states observed in the gas phase calculation. The complete assignment of electronic transitions is presented in Table S3. In water solvated system, the T_1_–T_4_ states are below the S_1_ state (Table 4), and all possess contributions from the d_Co_/π → π^*^ transition.

### Mechanism of photolysis

**Figure 4:**
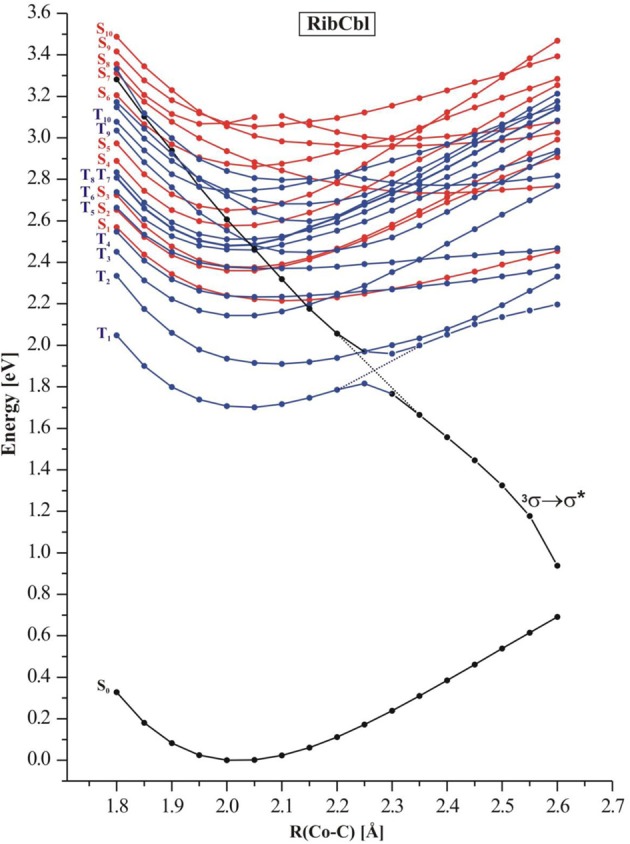
**Potential energy curves of the lowest-excited singlet (red) and triplet (blue) states of the RibCbl model complex along the Co-C bond stretch computed at TD-DFT/BP86/TZVP**. The triplet repulsive state is denoted as ^3^σ → σ^*^.

In order to simulate the photodissociation of AdoCbl, the Co-C_Rib_ bond in RibCbl was elongated to mimic the light induced bond scission. The length of the Co-C_Rib_ bond was systematically stretched with the increment of 0.05 Å in a range from 1.90 to 2.65 Å. For each constrained distance, the ground state geometry was optimized at the BP86/TZVP level, followed by TD-DFT calculations for both singlet and triplet states. Figure 4 depicts the potential energy curves of a manifold of excited states computed as a function of Co-C_Rib_ bond length.

The S_1_–S_5_ singlet states, which belong to α/β band, have non-repulsive character, and the gap between the S_1_ and higher states gets larger when the bond length increases. States denoted as S_2_ and S_3_ are parallel at short Co-C_Rib_ distance but cross at ~2.50 Å. On the other hand the S_4_ and S_5_ keep similar energy but visibly separate from the first group of transitions (S_1_–S_3_). However, at ~2.30 Å these two states show avoiding crossing, and the S_4_ approaches the S_2_ at long length bond region. The excited states higher in energy (S_6_–S_10_), display multiple crossings at even short Co-C distances. According to the TD-DFT data, low-lying singlet states do not participate in photodissociation because there is no ^1^(σ_Co−C_ → σ^*^_Co−C_) dominant. Most likely such a transition requires an energetically high excitation and none of singlet states with predominant ^1^(σ_Co−C_ → σ^*^_Co−C_) character are in a reasonable transition region. Additionally, it is expected that such states would not dissociate to the cob(II)alamin and ribosyl radicals, but rather to ionic fragments. However, the low-lying triplet states appear to have σ_Co−C_ → σ^*^_Co−C_ character in the achievable energy region. By connecting the energy points of σ_Co−C_-featured triplet states at different dissociation distances, the ^3^(σ_Co−C_ → σ^*^_Co−C_) state with repulsive character can be located (black curve in Figure 4). At 2.35 Å, this state becomes the lowest level in terms of energy, and displays an avoided crossing with T_1_ state, that typically accounts for d_Co_/π → π^*^ excitation. Together with an increasing Co-C_Rib_ distance, the energy of the triplet state drops dramatically above a Co-C bond distance of 2.40 Å, indicating that dissociation may not be properly described with a single-determinant wavefunction approximation at long Co-C_Rib_ distances (Kumar et al., 2011a,b). It is also reasonable to postulate that there is a conical intersection existing between the two states. Although the current study has limitations on the longer bond distances, it is expected that the photolysis happens at much shorter bond lengths, where ^3^(σ_Co−C_ → σ^*^_Co−C_) excitation is described properly. Further multi-configuration wave functions would be required to obtain a more detailed picture.

### Potential energy surfaces of low-lying excited states

**Figure 5:**
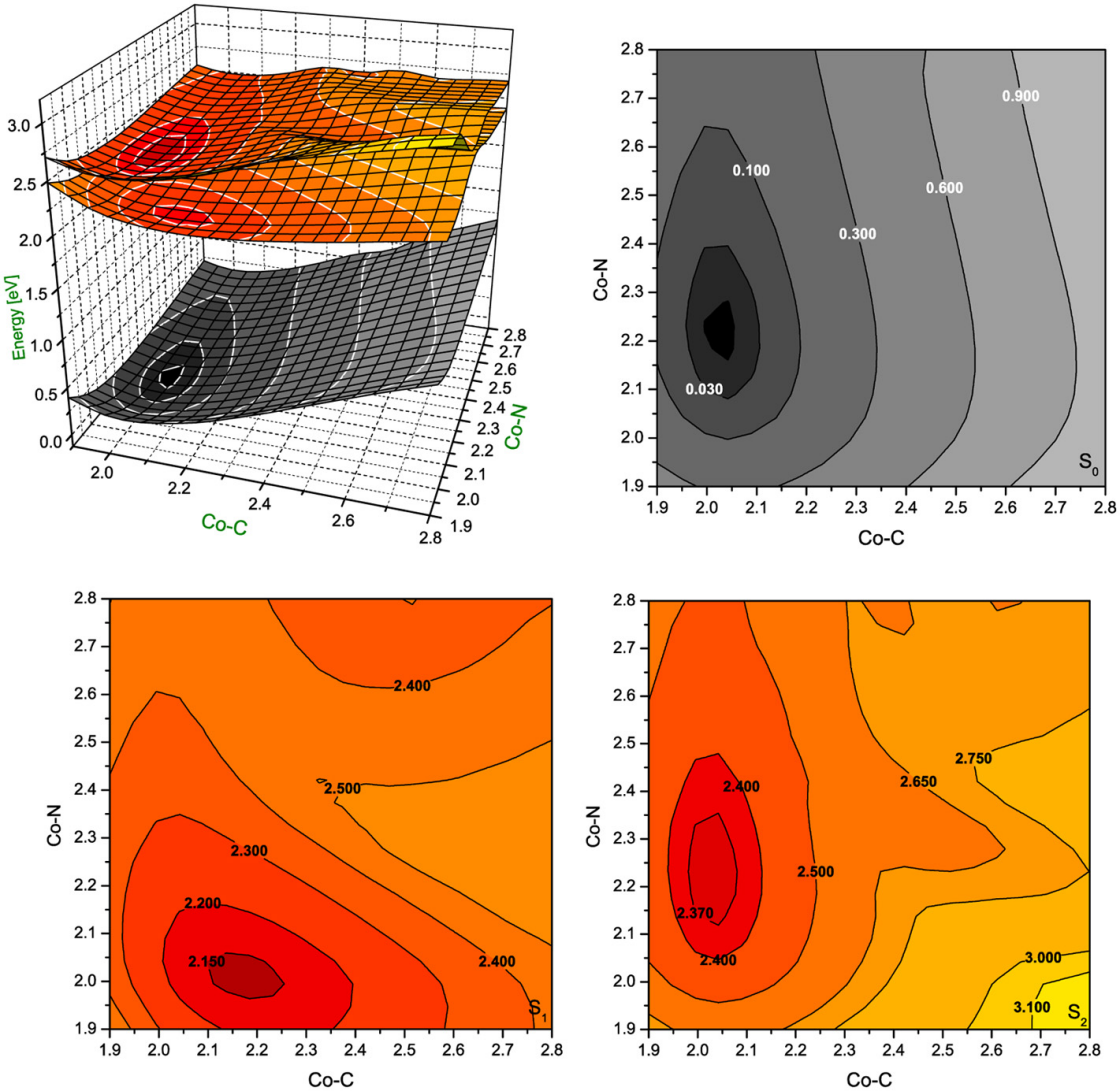
**Potential energy surfaces for singlet ground state and two lowest singlet excited states of RibCbl together with their vertical projections plotted as a function of axial bond lengths (expressed in Å) calculated in gas phase with BP86/TZVP**.

**Figure 6:**
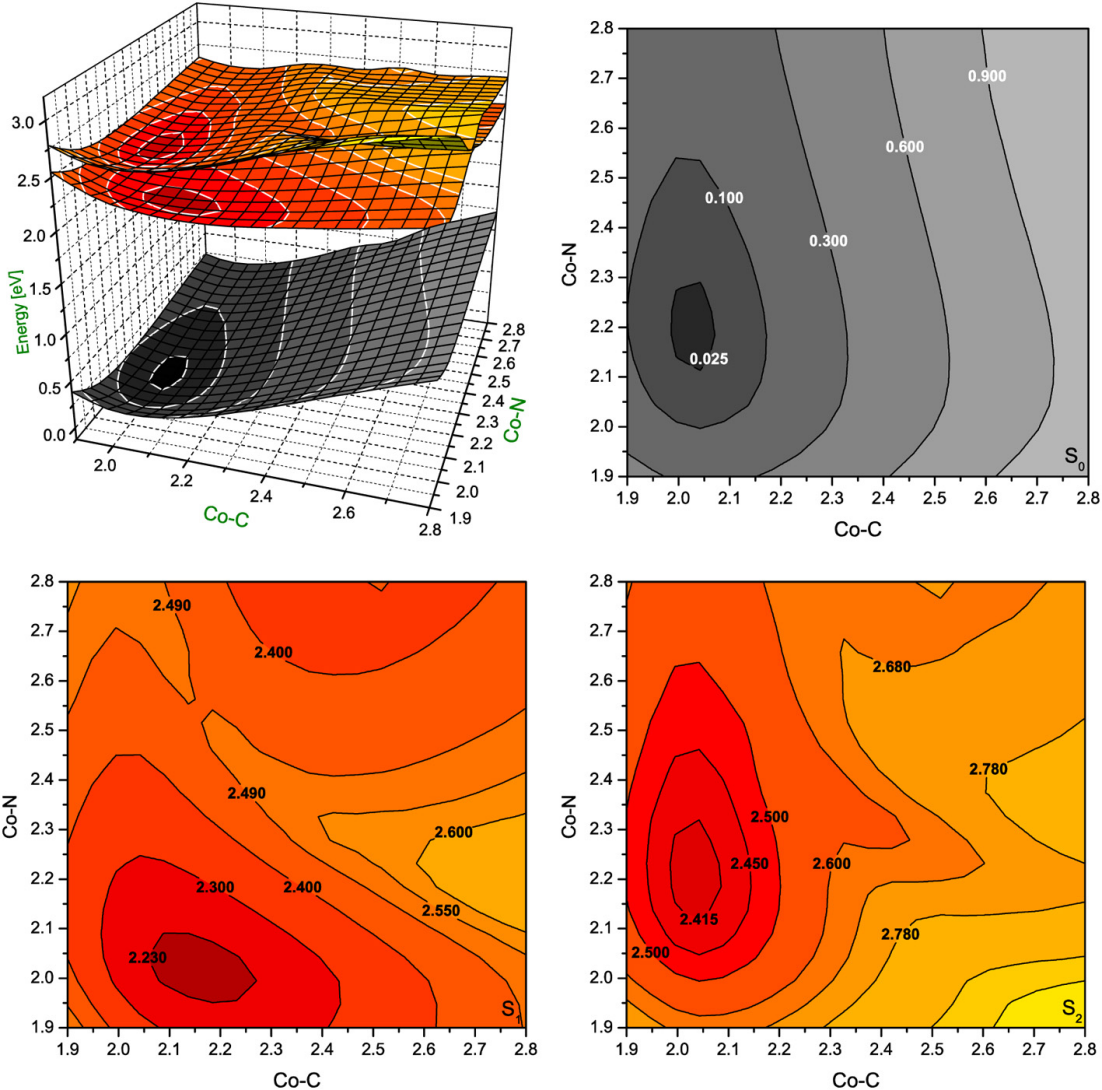
**Potential energy surfaces for singlet ground state and two lowest singlet excited states of RibCbl together with their vertical projections plotted as a function of axial bond lengths (expressed in Å) calculated in water solution (PCM) with BP86/TZVP**.

Since the photoexcitation of B_12_ derivatives involves primarily the structural changes of the axial ligand involved in N-Co-C bonding, this investigation was extended to the PESs computed as a function of both Co-C_Rib_ and Co-N_Im_ distances. The S_1_ and S_2_ PESs were constructed from the equilibrium structure of the RibCbl (S_0_ state), and systematically stretching the two axial bonds with an increment of 0.05 Å. Geometries at each grid point were optimized by BP86/TZVP, and the vertical excitations were calculated by TD-DFT at the BP86/TZVP level of theory (Figures 5, 6).

In gas phase PES contours (Figure 5), the S_0_ shows an equilibrium geometry with the lowest energy at 2.02 Å for Co-C_Rib_ and 2.21 Å for Co-N_Im_, while for the S_1_ PES, the minimum occurs at the longer Co-C_Rib_ bond (~2.17 Å) but shorter Co-N_Im_ (~2.00 Å) distance. According to pump-probe time resolved experiments, an intermediate state of AdoCbl is populated through photoelectronic excitation, leading to a partial mixture of adenosyl radical and cob(II)alamin. It is therefore reasonable to postulate that such an intermediate state can be identified as the S_1_, which according to TD-DFT calculations has the Co-C_Rib_ bond elongated and Co-N_Im_ shortened due to an electron density shift from the metal to the equatorial bonds of the corrin ring. The PES plot of the S_2_ is nearly parallel to the S_0_ surface. Estimates based on PES projections (Figure 5) predict the energy minimum of the S_2_ surface is located at Co-C_Rib_ ≈ 2.04 Å, and Co-N_Im_ ≈ 2.23 Å, ± 0.02 Å, compared to the equilibrium geometry. This indicates no significant change of axial bond distances occur from the S_0_ to S_2_ transition. If this is true, the energy difference between the S_0_ and S_2_ states are mainly attributed the geometry changes of the corrin ring. At this point, the distortion of the corrin ring, mainly due to π→π^*^ electronic transitions, increases the potential energy of the system. Since the geometry change in the S_1_ state involves Co-C_Rib_ bond stretching, it is likely that the S_1_ state is the most involved in the photolytic cleavage of Co-C, and thus the formation of the radical pair generated when the Co-C bond is stretched.

The PES of RibCbl in water retains nearly all of the general features of the gas phase PES (Figure 6). However, although the equilibrium geometry of RibCbl in water has similar axial bond lengths to those obtained in the gas phase calculation (with the exception of a slightly reduced Co-N bond distance of 0.02 Å), there remain subtle differences in energies between the S_0_ and S_1_, and S_0_ and S_2_ states. The energy gaps between S_0_ and S_1_, as well as S_0_ and S_2_, are larger for the solvated geometry than those of the gas phase. The vertical energy gaps between the minimum point of solvated ground state and the lowest two excited states are ~2.30 eV (S_0_–S_1_), and ~2.40 eV (S_0_–S_2_) respectively, while the vertical energy gaps in the gas phase calculation are ~2.20 eV (S_0_–S_1_) and ~2.35 eV (S_0_–S_2_). It can be reasoned that the larger vertical energy gaps from the ground state to the first two excited states in the PCM calculation, that the polar effect of solvent tends to increase the energies required for RibCbl excitation.

## Summary and conclusions

The purpose of this study was to explore the mechanisms of Co-C bond scission in AdoCbl upon light exposure using the TD-DFT method. To accomplish this we used a simplified RibCbl model to mimic the molecular features of photoexcited AdoCbl. The optimized RibCbl geometry at the DFT/BP86/TVZP level of theory was very close to the X-ray structure of AdoCbl with respect to the Co-C and Co-N_ax_ bond distances, within the deviation of 0.02 Å. Electronically excited states were calculated with TD-DFT, and both low-lying singlet and triplet states were analyzed. Calculated singlet states of RibCbl were used to generate electronic spectra that agree well with the experimental UV-Vis for AdoCbl, especially considering the characteristic low energy α/β band. In addition, PESs were generated as a function of both Co-C_Rib_ and Co-N_Im_ distances for singlet excited states using TD-DFT. The S_1_ state was identified as the key state of RibCbl photoexcitation, with contributions from both MLCT and SBLCT. Similar to the analysis of low-lying excited states of MeCbl, a repulsive ^3^(σ_Co−C_ → σ^*^_Co−C_) triplet state was found to facilitate photon induced dissociation of the Co-C bond in RibCbl.

### Conflict of interest statement

The authors declare that the research was conducted in the absence of any commercial or financial relationships that could be construed as a potential conflict of interest.

## Footnotes

^1^TURBOMOLE has been designed by the QuantumChemistryGroup, University of Karlsruhe, Germany, since 1988. The following members of the group have made contributions: Reinhart Ahlrichs, Michael Bär, Hans-Peter Baron, Rüdiger Bauernschmitt, Stephan Böcker, Nathan Crawford, Peter Deglmann, Michael Ehrig, Karin Eichkorn, Simon Elliott, Filipp Furche, Frank Haase, Marco Häser, Christof Hättig, Arnim Hellweg, Hans Horn, Christian Huber, Uwe Huniar, Marco Kattannek, Andreas Köhn, Christoph Kölmel, Markus Kollwitz, Klaus May, Paola Nava, Christian Ochsenfeld, Holger Öhm, Holger Patzelt, Dmitrij Rappoport, Oliver Rubner, Ansgar Schäfer, Uwe Schneider, Marek Sierka, Oliver Treutler, Barbara Unterreiner, Malte von Arnim, Florian Weigend, Patrick Weis, Horst Weiss. Available online at: http://www.turbomole.com.
