# Supplementary material for: TD-DFT insight into photodissociation of the Co-C bond in coenzyme B12
Source: Front Chem. 2014 Feb 5;1:41. doi: 10.3389/fchem.2013.00041 (PMC3982521; doi:10.3389/fchem.2013.00041)
Supplement: Supplementary file 1 [file Presentation1.PDF]

Supporting information

Table S1. The forty lowest singlet electronic excited states for model structure of RibCbl (Im-[Co^{III}(corrin)]-Rib^{+}) obtained from the TDDFT/BP86/TZVP calculations.

Table S2. The forty lowest triplet electronic excited states for model structure of RibCbl (Im-[Co^{III}(corrin)]-Rib^{+}) obtained from the TDDFT/BP86/TZVP calculations.

Table S3. The forty lowest singlet electronic excited states for model structure of RibCbl (Im-[Co^{III}(corrin)]-Rib^{+}) obtained from the TDDFT/BP86/TZVP calculations with use PCM/H_2O solvent model.

Table S4. The forty lowest triplet electronic excited states for model structure of RibCbl (Im-[Co^{III}(corrin)]-Rib^{+}) obtained from the TDDFT/BP86/TZVP calculations with use PCM/H_2O solvent model.

Table S5. MO energy and fragments contributions for model structure of RibCbl calculated in gas phase employing the DFT/BP86/TZVP level of theory.

Table S6. MO energy and fragments contributions for model structure of RibCbl calculated in water solution (PCM) employing the DFT/BP86/TZVP level of theory.

Figure S1. Molecular structure of RibCbl.

Figure S2. DFT–based isosurface plots of relevant MOs of gas phase Im-[Co^{III}(corrin)]-Rib^{+} obtained by DFT/BP86/TZVP level of theory. The MOLEKEL program was used to generate and visualize molecular orbitals.

Table S1. The forty lowest singlet electronic excited states for model structure of RibCbl (Im-[Co^{III}(corrin)]-Rib^+) obtained from the TDDFT/BP86/TZVP calculations.

	E(eV)	λ(nm)	*f*	Coeff.			Character
S_1	2.24	554.1	0.0082	74	143 → 145	H-1 → L	$\pi+d_{xz}/d_{z^2} \rightarrow \pi^*$
				18	142 → 145	H-2 → L	$d_{xz}+\pi \rightarrow \pi^*$
S_2	2.36	526.1	0.0261	74	144 → 145	H → L	$d_{yz}+\pi \rightarrow \pi^*$
				14	142 → 145	H-2 → L	$d_{xz}+\pi \rightarrow \pi^*$
S_3	2.37	522.1	0.0213	58	142 → 145	H-2 → L	$d_{xz}+\pi \rightarrow \pi^*$
				12	144 → 145	H → L	$d_{yz}+\pi \rightarrow \pi^*$
				12	141 → 145	H-3 → L	$n_{Rib}/\sigma_{Rib}+d_{x^2-y^2}+\pi \rightarrow \pi^*$
				12	143 → 145	H-1 → L	$\pi+d_{xz}/d_{z^2} \rightarrow \pi^*$
S_4	2.58	481.1	0.0026	61	140 → 145	H-4 → L	$d_{x^2-y^2} \rightarrow \pi^*$
				35	141 → 145	H-3 → L	$n_{Rib}/\sigma_{Rib}+d_{x^2-y^2}+\pi \rightarrow \pi^*$
S_5	2.65	467.2	0.0337	44	141 → 145	H-3 → L	$n_{Rib}/\sigma_{Rib}+d_{x^2-y^2}+\pi \rightarrow \pi^*$
				33	140 → 145	H-4 → L	$d_{x^2-y^2} \rightarrow \pi^*$
				10	143 → 145	H-1 → L	$\pi+d_{xz}/d_{z^2} \rightarrow \pi^*$
S_6	2.87	432.5	0.0186	65	144 → 146	H → L+1	$d_{yz}+\pi \rightarrow d_{xy}-n+\pi^*$
				12	143 → 146	H-1 → L+1	$\pi+d_{xz}/d_{z^2} \rightarrow d_{xy}-n+\pi^*$
S_7	2.95	421.0	0.0051	62	143 → 146	H-1 → L+1	$\pi+d_{xz}/d_{z^2} \rightarrow d_{xy}-n+\pi^*$
				13	144 → 146	H → L+1	$d_{yz}+\pi \rightarrow d_{xy}-n+\pi^*$
				9	144 → 148	H → L+3	$d_{yz}+\pi \rightarrow \sigma^*(d_{z^2})+ n$
S_8	3.04	408.2	0.0008	36	144 → 148	H → L+3	$d_{yz}+\pi \rightarrow \sigma^*(d_{z^2})+ n$
				27	144 → 147	H → L+2	$d_{yz}+\pi \rightarrow d_{xy}-n+\pi^*$
				18	139 → 145	H-5 → L	$n_{Rib}/\sigma_{Rib}+d_{xz}/d_{z^2}+\pi \rightarrow \pi^*$
S_9	3.05	406.1	0.0050	41	139 → 145	H-5 → L	$n_{Rib}/\sigma_{Rib}+d_{xz}/d_{z^2}+\pi \rightarrow \pi^*$
				31	144 → 148	H → L+3	$d_{yz}+\pi \rightarrow \sigma^*(d_{z^2})+ n$
				10	142 → 146	H-2 → L+1	$d_{xz}+\pi \rightarrow d_{xy}-n+\pi^*$
S_{10}	3.10	400.5	0.0269	43	144 → 147	H → L+2	$d_{yz}+\pi \rightarrow d_{xy}-n+\pi^*$
				28	139 → 145	H-5 → L	$n_{Rib}/\sigma_{Rib}+d_{xz}/d_{z^2}+\pi \rightarrow \pi^*$
S_{11}	3.13	396.3	0.0089	44	143 → 147	H-1 → L+2	$\pi+d_{xz}/d_{z^2} \rightarrow d_{xy}-n+\pi^*$
				12	138 → 145	H-6 → L	$\pi+ d_{yz} \rightarrow \pi^*$
				12	141 → 146	H-3 → L+1	$n_{Rib}/\sigma_{Rib}+d_{x^2-y^2}+\pi \rightarrow d_{xy}-n+\pi^*$
				8	143 → 148	H-1 → L+3	$\pi+d_{xz}/d_{z^2} \rightarrow \sigma^*(d_{z^2})+ n$
S_{12}	3.21	386.0	0.0313	62	143 → 148	H-1 → L+3	$\pi+d_{xz}/d_{z^2} \rightarrow \sigma^*(d_{z^2})+ n$
				11	142 → 146	H-2 → L+1	$d_{xz}+\pi \rightarrow d_{xy}-n+\pi^*$
				10	142 → 147	H-2 → L+2	$d_{xz}+\pi \rightarrow d_{xy}-n+\pi^*$
S_{13}	3.26	380.6	0.0036	36	141 → 146	H-3 → L+1	$n_{Rib}/\sigma_{Rib}+d_{x^2-y^2}+\pi \rightarrow d_{xy}-n+\pi^*$
				29	140 → 146	H-4 → L+1	$d_{x^2-y^2} \rightarrow d_{xy}-n+\pi^*$
S_{14}	3.27	379.5	0.0017	35	141 → 146	H-3 → L+1	$n_{Rib}/\sigma_{Rib}+d_{x^2-y^2}+\pi \rightarrow d_{xy}-n+\pi^*$
				24	142 → 148	H-2 → L+3	$d_{xz}+\pi \rightarrow \sigma^*(d_{z^2})+ n$
				14	140 → 146	H-4 → L+1	$d_{x^2-y^2} \rightarrow d_{xy}-n+\pi^*$
				9	142 → 146	H-2 → L+1	$d_{xz}+\pi \rightarrow d_{xy}-n+\pi^*$
S_{15}	3.31	374.3	0.0020	90	137 → 145	H-7 → L	$\pi_{Im} \rightarrow \pi^*$
S_{16}	3.37	367.7	0.0081	17	138 → 145	H-6 → L	$\pi+ d_{yz} \rightarrow \pi^*$
				50	141 → 147	H-3 → L+2	$n_{Rib}/\sigma_{Rib}+d_{x^2-y^2}+\pi \rightarrow d_{xy}-n+\pi^*$
S_{17}	3.43	361.9	0.0036	45	140 → 147	H-4 → L+2	$d_{x^2-y^2} \rightarrow d_{xy}-n+\pi^*$
				24	140 → 146	H-4 → L+1	$d_{x^2-y^2} \rightarrow d_{xy}-n+\pi^*$
S_{18}	3.47	357.8	0.0171	28	140 → 147	H-4 → L+2	$d_{x^2-y^2} \rightarrow d_{xy}-n+\pi^*$
				27	141 → 147	H-3 → L+2	$n_{Rib}/\sigma_{Rib}+d_{x^2-y^2}+\pi \rightarrow d_{xy}-n+\pi^*$
				9	142 → 146	H-2 → L+1	$d_{xz}+\pi \rightarrow d_{xy}-n+\pi^*$

S_{19}	3.52	352.0	0.0187	28	142 $\rightarrow$ 147	H-2 $\rightarrow$ L+2	$d_{xz}+\pi \rightarrow d_{xy}\text{-}n+\pi^*$
				16	141 $\rightarrow$ 148	H-3 $\rightarrow$ L+3	$n_{Rib}/\sigma_{Rib}+d_{x^2-y^2}+\pi \rightarrow \sigma^*(d_{z^2})+ n$
				15	142 $\rightarrow$ 146	H-2 $\rightarrow$ L+1	$d_{xz}+\pi \rightarrow d_{xy}\text{-}n+\pi^*$
S_{20}	3.56	348.0	0.0020	34	142 $\rightarrow$ 148	H-2 $\rightarrow$ L+3	$d_{xz}+\pi \rightarrow \sigma^*(d_{z^2})+ n$
				15	140 $\rightarrow$ 147	H-4 $\rightarrow$ L+2	$d_{x^2-y^2} \rightarrow d_{xy}\text{-}n+\pi^*$
S_{21}	3.60	344.2	0.0276	49	141 $\rightarrow$ 148	H-3 $\rightarrow$ L+3	$n_{Rib}/\sigma_{Rib}+d_{x^2-y^2}+\pi \rightarrow \sigma^*(d_{z^2})+ n$
				16	144 $\rightarrow$ 149	H $\rightarrow$ L+4	$d_{yz}+\pi \rightarrow \pi^*$
				12	140 $\rightarrow$ 148	H-4 $\rightarrow$ L+3	$d_{x^2-y^2} \rightarrow \sigma^*(d_{z^2})+ n$
S_{22}	3.63	341.2	0.0058	40	136 $\rightarrow$ 145	H-8 $\rightarrow$ L	$\sigma(d_{z^2})+n_{Rib}+\pi \rightarrow \pi^*$
				20	142 $\rightarrow$ 147	H-2 $\rightarrow$ L+2	$d_{xz}+\pi \rightarrow d_{xy}\text{-}n+\pi^*$
S_{23}	3.69	336.0	0.0009	41	135 $\rightarrow$ 145	H-9 $\rightarrow$ L	$n_{Rib}/\sigma_{Rib} \rightarrow \pi^*$
				15	136 $\rightarrow$ 145	H-8 $\rightarrow$ L	$\sigma(d_{z^2})+n_{Rib}+\pi \rightarrow \pi^*$
				12	144 $\rightarrow$ 149	H $\rightarrow$ L+4	$d_{yz}+\pi \rightarrow \pi^*$
S_{24}	3.71	334.0	0.0010	37	135 $\rightarrow$ 145	H-9 $\rightarrow$ L	$n_{Rib}/\sigma_{Rib} \rightarrow \pi^*$
				28	143 $\rightarrow$ 149	H-1 $\rightarrow$ L+4	$\pi+d_{xz}/d_{z^2} \rightarrow \pi^*$
				16	144 $\rightarrow$ 149	H $\rightarrow$ L+4	$d_{yz}+\pi \rightarrow \pi^*$
S_{25}	3.74	331.2	0.0426	42	139 $\rightarrow$ 146	H-5 $\rightarrow$ L+1	$n_{Rib}/\sigma_{Rib}+d_{xz}/d_{z^2}+\pi \rightarrow d_{xy}\text{-}n+\pi^*$
				29	144 $\rightarrow$ 149	H $\rightarrow$ L+4	$d_{yz}+\pi \rightarrow \pi^*$
				9	143 $\rightarrow$ 149	H-1 $\rightarrow$ L+4	$\pi+d_{xz}/d_{z^2} \rightarrow$
S_{26}	3.82	324.7	0.0166	30	139 $\rightarrow$ 146	H-5 $\rightarrow$ L+1	$n_{Rib}/\sigma_{Rib}+d_{xz}/d_{z^2}+\pi \rightarrow d_{xy}\text{-}n+\pi^*$
				27	143 $\rightarrow$ 149	H-1 $\rightarrow$ L+4	$\pi+d_{xz}/d_{z^2} \rightarrow \pi^*$
				14	134 $\rightarrow$ 145	H-10 $\rightarrow$ L	$\pi+ n_{Rib}/\sigma_{Rib}+d_{xz}/d_{z^2} \rightarrow \pi^*$
				11	142 $\rightarrow$ 149	H-2 $\rightarrow$ L+4	$d_{xz}+\pi \rightarrow \pi^*$
S_{27}	3.83	323.7	0.0766	14	139 $\rightarrow$ 146	H-5 $\rightarrow$ L+1	$n_{Rib}/\sigma_{Rib}+d_{xz}/d_{z^2}+\pi \rightarrow d_{xy}\text{-}n+\pi^*$
				10	138 $\rightarrow$ 145	H-6 $\rightarrow$ L	$\pi+ d_{yz} \rightarrow \pi^*$
				10	136 $\rightarrow$ 145	H-8 $\rightarrow$ L	$\sigma(d_{z^2})+n_{Rib}+\pi \rightarrow \pi^*$
				10	144 $\rightarrow$ 149	H $\rightarrow$ L+4	$d_{yz}+\pi \rightarrow \pi^*$
				9	142 $\rightarrow$ 149	H-2 $\rightarrow$ L+4	$d_{xz}+\pi \rightarrow \pi^*$
S_{28}	3.89	318.5	0.0066	91	144 $\rightarrow$ 150	H $\rightarrow$ L+5	$d_{yz}+\pi \rightarrow \pi^*_{Im}$
S_{29}	3.93	315.7	0.0672	62	142 $\rightarrow$ 149	H-2 $\rightarrow$ L+4	$d_{xz}+\pi \rightarrow \pi^*$
				11	134 $\rightarrow$ 145	H-10 $\rightarrow$ L	$\pi+ n_{Rib}/\sigma_{Rib}+d_{xz}/d_{z^2} \rightarrow \pi^*$
S_{30}	3.94	314.6	0.0192	79	139 $\rightarrow$ 147	H-5 $\rightarrow$ L+2	$n_{Rib}/\sigma_{Rib}+d_{xz}/d_{z^2}+\pi \rightarrow d_{xy}\text{-}n+\pi^*$
S_{31}	3.97	312.1	0.0120	39	138 $\rightarrow$ 146	H-6 $\rightarrow$ L+1	$\pi+ d_{yz} \rightarrow d_{xy}\text{-}n+\pi^*$
				25	134 $\rightarrow$ 145	H-10 $\rightarrow$ L	$\pi+ n_{Rib}/\sigma_{Rib}+d_{xz}/d_{z^2} \rightarrow \pi^*$
				8	143 $\rightarrow$ 150	H-1 $\rightarrow$ L+5	$\pi+d_{xz}/d_{z^2} \rightarrow \pi^*_{Im}$
S_{32}	4.01	309.1	0.0238	77	143 $\rightarrow$ 150	H-1 $\rightarrow$ L+5	$\pi+d_{xz}/d_{z^2} \rightarrow \pi^*_{Im}$
S_{33}	4.04	307.2	0.0060	83	137 $\rightarrow$ 146	H-7 $\rightarrow$ L+1	$\pi_{Im} \rightarrow d_{xy}\text{-}n+\pi^*$
S_{34}	4.04	307.0	0.0181	37	140 $\rightarrow$ 148	H-4 $\rightarrow$ L+3	$d_{x^2-y^2} \rightarrow \sigma^*(d_{z^2})+ n$
				11	134 $\rightarrow$ 145	H-10 $\rightarrow$ L	$\pi+ n_{Rib}/\sigma_{Rib}+d_{xz}/d_{z^2} \rightarrow \pi^*$
S_{35}	4.07	304.5	0.0029	74	141 $\rightarrow$ 149	H-3 $\rightarrow$ L+4	$n_{Rib}/\sigma_{Rib}+d_{x^2-y^2}+\pi \rightarrow \pi^*$
S_{36}	4.10	302.4	0.0056	81	140 $\rightarrow$ 149	H-4 $\rightarrow$ L+4	$d_{x^2-y^2} \rightarrow \pi^*$
S_{37}	4.14	299.2	0.0380	56	139 $\rightarrow$ 148	H-5 $\rightarrow$ L+3	$n_{Rib}/\sigma_{Rib}+d_{xz}/d_{z^2}+\pi \rightarrow \sigma^*(d_{z^2})+ n$
				12	142 $\rightarrow$ 150	H-2 $\rightarrow$ L+5	$d_{xz}+\pi \rightarrow \pi^*_{Im}$
S_{38}	4.19	296.1	0.0017	58	142 $\rightarrow$ 150	H-2 $\rightarrow$ L+5	$d_{xz}+\pi \rightarrow \pi^*_{Im}$
				22	137 $\rightarrow$ 147	H-7 $\rightarrow$ L+2	$\pi_{Im} \rightarrow d_{xy}\text{-}n+\pi^*$
S_{39}	4.20	295.4	0.0026	54	137 $\rightarrow$ 147	H-7 $\rightarrow$ L+2	$\pi_{Im} \rightarrow d_{xy}\text{-}n+\pi^*$
				27	142 $\rightarrow$ 150	H-2 $\rightarrow$ L+5	$d_{xz}+\pi \rightarrow \pi^*_{Im}$
				10	138 $\rightarrow$ 147	H-6 $\rightarrow$ L+2	$\pi+ d_{yz} \rightarrow d_{xy}\text{-}n+\pi^*$
S_{40}	4.23	293.4	0.0084	30	138 $\rightarrow$ 147	H-6 $\rightarrow$ L+2	$\pi+ d_{yz} \rightarrow d_{xy}\text{-}n+\pi^*$
				22	138 $\rightarrow$ 146	H-6 $\rightarrow$ L+1	$\pi+ d_{yz} \rightarrow d_{xy}\text{-}n+\pi^*$
				20	137 $\rightarrow$ 147	H-7 $\rightarrow$ L+2	$\pi_{Im} \rightarrow d_{xy}\text{-}n+\pi^*$

Table S2. The forty lowest triplet electronic excited states for model structure of RibCbl (Im-[Co^{III}(corrin)]-Rib^+) obtained from the TDDFT/BP86/TZVP calculations.

	E(eV)	λ(nm)	Coeff.			Character
T_1	1.72	720.0	49	144 → 145	H → L	$d_{yz}+\pi \rightarrow \pi^*$
T_2	1.94	639.9	48	143 → 145	H-1 → L	$\pi+d_{xz}/d_{z^2} \rightarrow \pi^*$
T_3	2.15	577.6	47	142 → 145	H-2 → L	$d_{xz}+\pi \rightarrow \pi^*$
T_4	2.25	551.8	35	144 → 146	H → L+1	$d_{yz}+\pi \rightarrow d_{xy}$-n+$\pi^*$
			10	144 → 147	H → L+2	$d_{yz}+\pi \rightarrow d_{xy}$-n+$\pi^*$
T_5	2.39	518.7	30	142 → 146	H-2 → L+1	$d_{xz}+\pi \rightarrow d_{xy}$-n+$\pi^*$
			8	142 → 147	H-2 → L+2	$d_{xz}+\pi \rightarrow d_{xy}$-n+$\pi^*$
T_6	2.47	501.4	28	141 → 145	H-3 → L	$n_{Rib}/\sigma_{Rib}+d_{x^2-y^2}+\pi \rightarrow \pi^*$
			18	140 → 145	H-4 → L	$d_{x^2-y^2} \rightarrow \pi^*$
T_7	2.48	499.6	26	140 → 145	H-4 → L	$d_{x^2-y^2} \rightarrow \pi^*$
			18	141 → 145	H-3 → L	$n_{Rib}/\sigma_{Rib}+d_{x^2-y^2}+\pi \rightarrow \pi^*$
T_8	2.52	491.2	29	140 → 146	H-4 → L+1	$d_{x^2-y^2} \rightarrow d_{xy}$-n+$\pi^*$
			12	140 → 147	H-4 → L+2	$d_{x^2-y^2} \rightarrow d_{xy}$-n+$\pi^*$
			5	141 → 146	H-3 → L+1	$n_{Rib}/\sigma_{Rib}+d_{x^2-y^2}+\pi \rightarrow d_{xy}$-n+$\pi^*$
T_9	2.55	487.0	15	144 → 148	H → L+3	$d_{yz}+\pi \rightarrow \sigma^*(d_{z^2})$+ n
			9	144 → 147	H → L+2	$d_{yz}+\pi \rightarrow d_{xy}$-n+$\pi^*$
			8	143 → 146	H-1 → L+1	$\pi+d_{xz}/d_{z^2} \rightarrow d_{xy}$-n+$\pi^*$
			5	144 → 146	H → L+1	$d_{yz}+\pi \rightarrow d_{xy}$-n+$\pi^*$
T_{10}	2.58	479.8	11	143 → 148	H-1 → L+3	$\pi+d_{xz}/d_{z^2} \rightarrow \sigma^*(d_{z^2})$+ n
			10	143 → 146	H-1 → L+1	$\pi+d_{xz}/d_{z^2} \rightarrow d_{xy}$-n+$\pi^*$
			6	144 → 148	H → L+3	$d_{yz}+\pi \rightarrow \sigma^*(d_{z^2})$+ n
			6	143 → 147	H-1 → L+2	$\pi+d_{xz}/d_{z^2} \rightarrow d_{xy}$-n+$\pi^*$
			4	144 → 147	H → L+2	$d_{yz}+\pi \rightarrow d_{xy}$-n+$\pi^*$
			4	142 → 147	H-2 → L+2	$d_{xz}+\pi \rightarrow d_{xy}$-n+$\pi^*$
T_{11}	2.72	456.5	21	142 → 148	H-2 → L+3	$d_{xz}+\pi \rightarrow \sigma^*(d_{z^2})$+ n
			10	142 → 147	H-2 → L+2	$d_{xz}+\pi \rightarrow d_{xy}$-n+$\pi^*$
			5	143 → 148	H-1 → L+3	$\pi+d_{xz}/d_{z^2} \rightarrow \sigma^*(d_{z^2})$+ n
T_{12}	2.73	454.4	22	143 → 146	H-1 → L+1	$\pi+d_{xz}/d_{z^2} \rightarrow d_{xy}$-n+$\pi^*$
			16	143 → 148	H-1 → L+3	$\pi+d_{xz}/d_{z^2} \rightarrow \sigma^*(d_{z^2})$+ n
T_{13}	2.75	450.3	21	144 → 147	H → L+2	$d_{yz}+\pi \rightarrow d_{xy}$-n+$\pi^*$
			18	144 → 148	H → L+3	$d_{yz}+\pi \rightarrow \sigma^*(d_{z^2})$+ n
			6	144 → 146	H → L+1	$d_{yz}+\pi \rightarrow d_{xy}$-n+$\pi^*$
T_{14}	2.83	438.1	25	143 → 147	H-1 → L+2	$\pi+d_{xz}/d_{z^2} \rightarrow d_{xy}$-n+$\pi^*$
			13	142 → 148	H-2 → L+3	$d_{xz}+\pi \rightarrow \sigma^*(d_{z^2})$+ n
			5	138 → 145	H-6 → L	$\pi+ d_{yz} \rightarrow \pi^*$
T_{15}	2.88	430.5	24	142 → 147	H-2 → L+2	$d_{xz}+\pi \rightarrow d_{xy}$-n+$\pi^*$
			11	142 → 148	H-2 → L+3	$d_{xz}+\pi \rightarrow \sigma^*(d_{z^2})$+ n
			7	143 → 147	H-1 → L+2	$\pi+d_{xz}/d_{z^2} \rightarrow d_{xy}$-n+$\pi^*$
			5	142 → 146	H-2 → L+1	$d_{xz}+\pi \rightarrow d_{xy}$-n+$\pi^*$
T_{16}	2.99	414.9	21	138 → 145	H-6 → L	$\pi+ d_{yz} \rightarrow \pi^*$
			20	139 → 145	H-5 → L	$n_{Rib}/\sigma_{Rib}+d_{xz}/d_{z^2}+\pi \rightarrow \pi^*$
T_{17}	3.03	409.6	28	139 → 145	H-5 → L	$n_{Rib}/\sigma_{Rib}+d_{xz}/d_{z^2}+\pi \rightarrow \pi^*$
			16	138 → 145	H-6 → L	$\pi+ d_{yz} \rightarrow \pi^*$
T_{18}	3.04	407.4	25	140 → 148	H-4 → L+3	$d_{x^2-y^2} \rightarrow \sigma^*(d_{z^2})$+ n
			10	140 → 147	H-4 → L+2	$d_{x^2-y^2} \rightarrow d_{xy}$-n+$\pi^*$
			5	141 → 148	H-3 → L+3	$n_{Rib}/\sigma_{Rib}+d_{x^2-y^2}+\pi \rightarrow \sigma^*(d_{z^2})$+ n
			4	140 → 146	H-4 → L+1	$d_{x^2-y^2} \rightarrow d_{xy}$-n+$\pi^*$
T_{19}	3.20	387.0	41	141 → 146	H-3 → L+1	$n_{Rib}/\sigma_{Rib}+d_{x^2-y^2}+\pi \rightarrow d_{xy}$-n+$\pi^*$

			7	140 → 146	H-4 → L+1	$d_{x^2-y^2} \rightarrow d_{xy}$-n+$\pi^*$
T_{20}	3.30	375.2	46	137 → 145	H-7 → L	$\pi_{Im} \rightarrow \pi^*$
T_{21}	3.32	373.1	14	140 → 147	H-4 → L+2	$d_{x^2-y^2} \rightarrow d_{xy}$-n+$\pi^*$
			13	140 → 148	H-4 → L+3	$d_{x^2-y^2} \rightarrow \sigma^*(d_{z^2})$+ n
			8	140 → 146	H-4 → L+1	$d_{x^2-y^2} \rightarrow d_{xy}$-n+$\pi^*$
			8	141 → 147	H-3 → L+2	n_{Rib}/σ_{Rib}+ $d_{x^2-y^2}+\pi \rightarrow d_{xy}$-n+$\pi^*$
T_{22}	3.36	368.7	32	141 → 147	H-3 → L+2	n_{Rib}/σ_{Rib}+ $d_{x^2-y^2}+\pi \rightarrow d_{xy}$-n+$\pi^*$
			9	140 → 147	H-4 → L+2	$d_{x^2-y^2} \rightarrow d_{xy}$-n+$\pi^*$
			6	144 → 149	H → L+4	$d_{yz}+\pi \rightarrow \pi^*$
T_{23}	3.41	363.4	32	144 → 149	H → L+4	$d_{yz}+\pi \rightarrow \pi^*$
			6	143 → 149	H-1 → L+4	$\pi+d_{xz}/d_{z^2} \rightarrow \pi^*$
			4	141 → 147	H-3 → L+2	n_{Rib}/σ_{Rib}+ $d_{x^2-y^2}+\pi \rightarrow d_{xy}$-n+$\pi^*$
T_{24}	3.42	362.1	27	143 → 149	H-1 → L+4	$\pi+d_{xz}/d_{z^2} \rightarrow \pi^*$
			8	144 → 149	H → L+4	$d_{yz}+\pi \rightarrow \pi^*$
T_{25}	3.47	357.7	37	141 → 148	H-3 → L+3	n_{Rib}/σ_{Rib}+ $d_{x^2-y^2}+\pi \rightarrow \sigma^*(d_{z^2})$+ n
			9	140 → 148	H-4 → L+3	$d_{x^2-y^2} \rightarrow \sigma^*(d_{z^2})$+ n
T_{26}	3.55	349.2	31	136 → 145	H-8 → L	$\sigma(d_{z^2})+n_{Rib}+\pi \rightarrow \pi^*$
			11	143 → 149	H-1 → L+4	$\pi+d_{xz}/d_{z^2} \rightarrow \pi^*$
T_{27}	3.62	342.4	15	135 → 145	H-9 → L	$n_{Rib}/\sigma_{Rib} \rightarrow \pi^*$
			13	136 → 145	H-8 → L	$\sigma(d_{z^2})+n_{Rib}+\pi \rightarrow \pi^*$
			10	134 → 145	H-10 → L	$\pi+ n_{Rib}/\sigma_{Rib}+d_{xz}/d_{z^2} \rightarrow \pi^*$
T_{28}	3.70	335.3	43	139 → 146	H-5 → L+1	$n_{Rib}/\sigma_{Rib}+d_{xz}/d_{z^2}+\pi \rightarrow d_{xy}$-n+$\pi^*$
T_{29}	3.72	333.6	20	135 → 145	H-9 → L	$n_{Rib}/\sigma_{Rib} \rightarrow \pi^*$
			16	142 → 149	H-2 → L+4	$d_{xz}+\pi \rightarrow \pi^*$
			10	138 → 146	H-6 → L+1	$\pi+ d_{yz} \rightarrow d_{xy}$-n+$\pi^*$
T_{30}	3.75	330.6	24	142 → 149	H-2 → L+4	$d_{xz}+\pi \rightarrow \pi^*$
			19	138 → 146	H-6 → L+1	$\pi+ d_{yz} \rightarrow d_{xy}$-n+$\pi^*$
T_{31}	3.77	328.5	22	134 → 145	H-10 → L	$\pi+ n_{Rib}/\sigma_{Rib}+d_{xz}/d_{z^2} \rightarrow \pi^*$
			11	138 → 146	H-6 → L+1	$\pi+ d_{yz} \rightarrow d_{xy}$-n+$\pi^*$
			6	135 → 145	H-9 → L	$n_{Rib}/\sigma_{Rib} \rightarrow \pi^*$
T_{32}	3.82	325.0	17	139 → 148	H-5 → L+3	$n_{Rib}/\sigma_{Rib}+d_{xz}/d_{z^2}+\pi \rightarrow \sigma^*(d_{z^2})$+ n
			12	139 → 147	H-5 → L+2	$n_{Rib}/\sigma_{Rib}+d_{xz}/d_{z^2}+\pi \rightarrow d_{xy}$-n+$\pi^*$
T_{33}	3.89	318.9	48	144 → 150	H → L+5	$d_{yz}+\pi \rightarrow \pi^*_{Im}$
T_{34}	3.90	317.9	36	139 → 147	H-5 → L+2	$n_{Rib}/\sigma_{Rib}+d_{xz}/d_{z^2}+\pi \rightarrow d_{xy}$-n+$\pi^*$
			8	139 → 148	H-5 → L+3	$n_{Rib}/\sigma_{Rib}+d_{xz}/d_{z^2}+\pi \rightarrow \sigma^*(d_{z^2})$+ n
T_{35}	3.94	314.7	37	143 → 150	H-1 → L+5	$\pi+d_{xz}/d_{z^2} \rightarrow \pi^*_{Im}$
			9	138 → 147	H-6 → L+2	$\pi+ d_{yz} \rightarrow d_{xy}$-n+$\pi^*$
T_{36}	3.95	314.0	28	138 → 147	H-6 → L+2	$\pi+ d_{yz} \rightarrow d_{xy}$-n+$\pi^*$
			12	143 → 150	H-1 → L+5	$\pi+d_{xz}/d_{z^2} \rightarrow \pi^*_{Im}$
T_{37}	4.01	309.0	46	137 → 146	H-7 → L+1	$\pi_{Im} \rightarrow d_{xy}$-n+$\pi^*$
T_{38}	4.02	308.7	28	138 → 148	H-6 → L+3	$\pi+ d_{yz} \rightarrow \sigma^*(d_{z^2})$+ n
			12	141 → 149	H-3 → L+4	n_{Rib}/σ_{Rib}+ $d_{x^2-y^2}+\pi \rightarrow \pi^*$
			7	139 → 148	H-5 → L+3	$n_{Rib}/\sigma_{Rib}+d_{xz}/d_{z^2}+\pi \rightarrow \sigma^*(d_{z^2})$+ n
T_{39}	4.03	307.3	35	141 → 149	H-3 → L+4	n_{Rib}/σ_{Rib}+ $d_{x^2-y^2}+\pi \rightarrow \pi^*$
			10	138 → 148	H-6 → L+3	$\pi+ d_{yz} \rightarrow \sigma^*(d_{z^2})$+ n
T_{40}	4.07	304.7	49	140 → 149	H-4 → L+4	$d_{x^2-y^2} \rightarrow \pi^*$

Table S3. The forty lowest singlet electronic excited states for model structure of RibCbl (Im-[Co^{III}(corrin)]-Rib^+) obtained from the TDDFT/BP86/TZVP calculations with use PCM/H_2O solvent model.

	E(eV)	λ(nm)	*f*	Coeff.			Character
S_1	2.28	543.4	0.0150	27	142 → 145	H-2 → L	$d_{xz}+\pi \rightarrow \pi^*$
				56	143 → 145	H-1 → L	$\pi+d_{yz}/d_{z^2} \rightarrow \pi^*$
				15	144 → 145	H → L	$d_{yz}+\pi \rightarrow \pi^*$
S_2	2.39	518.9	0.0353	74	144 → 145	H → L	$d_{yz}+\pi \rightarrow \pi^*$
S_3	2.45	506.4	0.0597	61	142 → 145	H-2 → L	$d_{xz}+\pi \rightarrow \pi^*$
				29	143 → 145	H-1 → L	$\pi+d_{yz}/d_{z^2} \rightarrow \pi^*$
S_4	2.62	473.5	0.0024	97	141 → 145	H-3 → L	$d_{x^2-y^2} \rightarrow \pi^*$
S_5	2.86	434.0	0.0043	62	144 → 146	H → L+1	$d_{yz}+\pi \rightarrow d_{xy}\text{-}n+\pi^*$
				16	140 → 145	H-4 → L	$n_{Rib}/\sigma_{Rib} \rightarrow \pi^*$
S_6	2.90	427.7	0.0235	62	140 → 145	H-4 → L	$n_{Rib}/\sigma_{Rib} \rightarrow \pi^*$
				23	143 → 146	H-1 → L+1	$\pi+d_{yz}/d_{z^2} \rightarrow d_{xy}\text{-}n+\pi^*$
S_7	2.94	421.8	0.0186	53	143 → 146	H-1 → L+1	$\pi+d_{yz}/d_{z^2} \rightarrow d_{xy}\text{-}n+\pi^*$
				14	140 → 145	H-4 → L	$n_{Rib}/\sigma_{Rib} \rightarrow \pi^*$
				14	144 → 146	H → L+1	$d_{yz}+\pi \rightarrow d_{xy}\text{-}n+\pi^*$
S_8	3.02	410.0	0.0053	59	144 → 148	H → L+3	$d_{yz}+\pi \rightarrow \sigma^*(d_{z^2})+ n$
				20	144 → 147	H → L+2	$d_{yz}+\pi \rightarrow d_{xy}\text{-}n+\pi^*$
S_9	3.09	401.4	0.0185	55	144 → 147	H → L+2	$d_{yz}+\pi \rightarrow d_{xy}\text{-}n+\pi^*$
				19	144 → 148	H → L+3	$d_{yz}+\pi \rightarrow \sigma^*(d_{z^2})+ n$
S_{10}	3.16	392.7	0.0112	35	139 → 145	H-5 → L	$\pi_{Im} \rightarrow \pi^*$
				33	143 → 148	H-1 → L+3	$\pi+d_{yz}/d_{z^2} \rightarrow \sigma^*(d_{z^2})+ n$
				15	143 → 147	H-1 → L+2	$\pi+d_{yz}/d_{z^2} \rightarrow d_{xy}\text{-}n+\pi^*$
				9	138 → 145	H-6 → L	$\pi+ d_{yz} \rightarrow \pi^*$
S_{11}	3.18	390.1	0.0093	55	139 → 145	H-5 → L	$\pi_{Im} \rightarrow \pi^*$
				20	138 → 145	H-6 → L	$\pi+ d_{yz} \rightarrow \pi^*$
				9	143 → 148	H-1 → L+3	$\pi+d_{yz}/d_{z^2} \rightarrow \sigma^*(d_{z^2})+ n$
S_{12}	3.19	388.5	0.0308	40	143 → 148	H-1 → L+3	$\pi+d_{yz}/d_{z^2} \rightarrow \sigma^*(d_{z^2})+ n$
				33	143 → 147	H-1 → L+2	$\pi+d_{yz}/d_{z^2} \rightarrow d_{xy}\text{-}n+\pi^*$
				9	142 → 147	H-2 → L+2	$d_{xz}+\pi \rightarrow d_{xy}\text{-}n+\pi^*$
S_{13}	3.27	378.7	0.0010	39	141 → 146	H-3 → L+1	$d_{x^2-y^2} \rightarrow d_{xy}\text{-}n+\pi^*$
				28	142 → 148	H-2 → L+3	$d_{xz}+\pi \rightarrow \sigma^*(d_{z^2})+ n$
				14	142 → 146	H-2 → L+1	$d_{xz}+\pi \rightarrow d_{xy}\text{-}n+\pi^*$
S_{14}	3.35	370.3	0.0280	78	137 → 145	H-7 → L	$n_{Rib}/\sigma_{Rib}+d_{yz}/d_{z^2}+\pi \rightarrow \pi^*$
				11	138 → 145	H-6 → L	$\pi+ d_{yz} \rightarrow \pi^*$
S_{15}	3.43	361.5	0.0167	35	141 → 146	H-3 → L+1	$d_{x^2-y^2} \rightarrow d_{xy}\text{-}n+\pi^*$
				23	142 → 146	H-2 → L+1	$d_{xz}+\pi \rightarrow d_{xy}\text{-}n+\pi^*$
				11	141 → 147	H-3 → L+2	$d_{x^2-y^2} \rightarrow d_{xy}\text{-}n+\pi^*$
				9	143 → 147	H-1 → L+2	$\pi+d_{yz}/d_{z^2} \rightarrow d_{xy}\text{-}n+\pi^*$
S_{16}	3.47	357.2	0.0331	45	141 → 147	H-3 → L+2	$d_{x^2-y^2} \rightarrow d_{xy}\text{-}n+\pi^*$
				14	142 → 148	H-2 → L+3	$d_{xz}+\pi \rightarrow \sigma^*(d_{z^2})+ n$
				10	138 → 145	H-6 → L	$\pi+ d_{yz} \rightarrow \pi^*$
S_{17}	3.52	352.2	0.0166	25	140 → 146	H-4 → L+1	$n_{Rib}/\sigma_{Rib} \rightarrow d_{xy}\text{-}n+\pi^*$
				18	141 → 147	H-3 → L+2	$d_{x^2-y^2} \rightarrow d_{xy}\text{-}n+\pi^*$
				15	138 → 145	H-6 → L	$\pi+ d_{yz} \rightarrow \pi^*$
				10	141 → 148	H-3 → L+3	$d_{x^2-y^2} \rightarrow \sigma^*(d_{z^2})+ n$
				10	142 → 146	H-2 → L+1	$d_{xz}+\pi \rightarrow d_{xy}\text{-}n+\pi^*$
S_{18}	3.56	348.4	0.0092	35	140 → 146	H-4 → L+1	$n_{Rib}/\sigma_{Rib} \rightarrow d_{xy}\text{-}n+\pi^*$
				24	142 → 148	H-2 → L+3	$d_{xz}+\pi \rightarrow \sigma^*(d_{z^2})+ n$

				10	141 → 148	H-3 → L+3	$d_{x2-y2} \to \sigma^*(d_{z2})$+ n
				9	141 → 147	H-3 → L+2	$d_{x2-y2} \to d_{xy}$-n+π^*
S_{19}	3.57	347.0	0.0093	44	142 → 147	H-2 → L+2	$d_{xz}+\pi \to d_{xy}$-n+π^*
				13	140 → 146	H-4 → L+1	$n_{Rib}/\sigma_{Rib} \to d_{xy}$-n+$\pi^*$
				13	142 → 146	H-2 → L+1	$d_{xz}+\pi \to d_{xy}$-n+π^*
S_{20}	3.65	339.2	0.0189	21	140 → 147	H-4 → L+2	$n_{Rib}/\sigma_{Rib} \to d_{xy}$-n+$\pi^*$
				17	140 → 146	H-4 → L+1	$n_{Rib}/\sigma_{Rib} \to d_{xy}$-n+$\pi^*$
				12	138 → 145	H-6 → L	π+ $d_{yz} \to \pi^*$
				12	142 → 148	H-2 → L+3	$d_{xz}+\pi \to \sigma^*(d_{z2})$+ n
				9	142 → 147	H-2 → L+2	$d_{xz}+\pi \to d_{xy}$-n+π^*
S_{21}	3.73	332.3	0.0245	72	144 → 149	H → L+4	$d_{yz}+\pi \to \pi^*$
				15	140 → 147	H-4 → L+2	$n_{Rib}/\sigma_{Rib} \to d_{xy}$-n+$\pi^*$
S_{22}	3.77	328.8	0.0009	54	143 → 149	H-1 → L+4	$\pi+d_{yz}/d_{z2} \to \pi^*$
				35	136 → 145	H-8 → L	$\pi+n_{Rib} \to \pi^*$
S_{23}	3.83	324.0	0.0949	45	140 → 147	H-4 → L+2	$n_{Rib}/\sigma_{Rib} \to d_{xy}$-n+$\pi^*$
				8	138 → 145	H-6 → L	π+ $d_{yz} \to \pi^*$
S_{24}	3.86	320.8	0.0132	88	139 → 146	H-5 → L+1	$\pi_{Im} \to d_{xy}$-n+π^*
S_{25}	3.90	318.3	0.0406	70	140 → 148	H-4 → L+3	$n_{Rib}/\sigma_{Rib} \to \sigma^*(d_{z2})$+ n
S_{26}	3.92	316.2	0.0289	54	142 → 149	H-2 → L+4	$d_{xz}+\pi \to \pi^*$
				9	135 → 145	H-9 → L	$\sigma(d_{z2})+n_{Rib}+\pi \to \pi^*$
				9	136 → 145	H-8 → L	$\pi+n_{Rib} \to \pi^*$
S_{27}	3.96	312.9	0.0356	42	138 → 146	H-6 → L+1	π+ $d_{yz} \to d_{xy}$-n+π^*
				17	136 → 145	H-8 → L	$\pi+n_{Rib} \to \pi^*$
				9	142 → 149	H-2 → L+4	$d_{xz}+\pi \to \pi^*$
				9	135 → 145	H-9 → L	$\sigma(d_{z2})+n_{Rib}+\pi \to \pi^*$
S_{28}	4.00	310.3	0.0817	54	135 → 145	H-9 → L	$\sigma(d_{z2})+n_{Rib}+\pi \to \pi^*$
				24	142 → 149	H-2 → L+4	$d_{xz}+\pi \to \pi^*$
S_{29}	4.04	306.7	0.0041	48	141 → 148	H-3 → L+3	$d_{x2-y2} \to \sigma^*(d_{z2})$+ n
				10	139 → 148	H-5 → L+3	$\pi_{Im} \to \sigma^*(d_{z2})$+ n
				8	141 → 147	H-3 → L+2	$d_{x2-y2} \to d_{xy}$-n+π^*
S_{30}	4.05	305.9	0.0020	78	139 → 147	H-5 → L+2	$\pi_{Im} \to d_{xy}$-n+π^*
				11	138 → 147	H-6 → L+2	π+ $d_{yz} \to d_{xy}$-n+π^*
S_{31}	4.09	303.3	0.0275	67	137 → 146	H-7 → L+1	$n_{Rib}/\sigma_{Rib}+d_{yz}/d_{z2}+\pi \to d_{xy}$-n+$\pi^*$
S_{32}	4.11	301.3	0.0014	97	144 → 150	H → L+5	$d_{yz}+\pi \to \pi^*_{Im}$
S_{33}	4.12	300.7	0.0038	66	139 → 148	H-5 → L+3	$\pi_{Im} \to \sigma^*(d_{z2})$+ n
				10	138 → 148	H-6 → L+3	π+ $d_{yz} \to \sigma^*(d_{z2})$+ n
				10	134 → 145	H-10 → L	$n_{Rib}/\sigma_{Rib} \to \pi^*$
S_{34}	4.13	300.0	0.0025	74	134 → 145	H-10 → L	$n_{Rib}/\sigma_{Rib} \to \pi^*$
				10	139 → 148	H-5 → L+3	$\pi_{Im} \to \sigma^*(d_{z2})$+ n
S_{35}	4.14	299.7	0.0009	78	141 → 149	H-3 → L+4	$d_{x2-y2} \to \pi^*$
				11	134 → 145	H-10 → L	$n_{Rib}/\sigma_{Rib} \to \pi^*$
S_{36}	4.18	296.3	0.0112	25	138 → 147	H-6 → L+2	π+ $d_{yz} \to d_{xy}$-n+π^*
				18	137 → 147	H-7 → L+2	$n_{Rib}/\sigma_{Rib}+d_{yz}/d_{z2}+\pi \to d_{xy}$-n+$\pi^*$
				15	138 → 146	H-6 → L+1	π+ $d_{yz} \to d_{xy}$-n+π^*
S_{37}	4.21	294.4	0.0085	87	143 → 150	H-1 → L+5	$\pi+d_{yz}/d_{z2} \to \pi^*_{Im}$
S_{38}	4.26	291.1	0.0275	30	138 → 148	H-6 → L+3	π+ $d_{yz} \to \sigma^*(d_{z2})$+ n
				15	137 → 147	H-7 → L+2	$n_{Rib}/\sigma_{Rib}+d_{yz}/d_{z2}+\pi \to d_{xy}$-n+$\pi^*$
				13	138 → 147	H-6 → L+2	π+ $d_{yz} \to d_{xy}$-n+π^*
S_{39}	4.29	289.0	0.0172	37	137 → 147	H-7 → L+2	$n_{Rib}/\sigma_{Rib}+d_{yz}/d_{z2}+\pi \to d_{xy}$-n+$\pi^*$
				24	138 → 148	H-6 → L+3	π+ $d_{yz} \to \sigma^*(d_{z2})$+ n
S_{40}	4.36	284.3	0.0075	65	140 → 149	H-4 → L+4	$n_{Rib}/\sigma_{Rib} \to \pi^*$
				9	138 → 147	H-6 → L+2	π+ $d_{yz} \to d_{xy}$-n+π^*
				8	138 → 148	H-6 → L+3	π+ $d_{yz} \to \sigma^*(d_{z2})$+ n
				8	137 → 147	H-7 → L+2	$n_{Rib}/\sigma_{Rib}+d_{yz}/d_{z2}+\pi \to d_{xy}$-n+$\pi^*$

Table S4. The forty lowest triplet electronic excited states for model structure of RibCbl (Im-[Co^{III}(corrin)]-Rib^+) obtained from the TDDFT/BP86/TZVP calculations with use PCM/H_2O solvent model.

	E(eV)	λ(nm)	Coeff.			Character
T_1	1.75	708.3	48	144 → 145	H → L	$d_{yz}+\pi \to \pi^*$
T_2	1.94	637.8	47	143 → 145	H-1 → L	$\pi+d_{yz}/d_{z^2} \to \pi^*$
T_3	2.19	567.1	45	142 → 145	H-2 → L	$d_{xz}+\pi \to \pi^*$
T_4	2.26	548.4	33	144 → 146	H → L+1	$d_{yz}+\pi \to d_{xy}$-n+π^*
			7	144 → 147	H → L+2	$d_{yz}+\pi \to d_{xy}$-n+π^*
T_5	2.40	516.3	34	142 → 146	H-2 → L+1	$d_{xz}+\pi \to d_{xy}$-n+π^*
			6	142 → 147	H-2 → L+2	$d_{xz}+\pi \to d_{xy}$-n+π^*
T_6	2.50	496.6	27	141 → 145	H-3 → L	$d_{x^2-y^2} \to \pi^*$
			15	141 → 146	H-3 → L+1	$d_{x^2-y^2} \to d_{xy}$-n+π^*
			5	141 → 147	H-3 → L+2	$d_{x^2-y^2} \to d_{xy}$-n+π^*
T_7	2.52	492.7	12	144 → 147	H → L+2	$d_{yz}+\pi \to d_{xy}$-n+π^*
			17	144 → 148	H → L+3	$d_{yz}+\pi \to \sigma^*(d_{z^2})$+ n
			5	141 → 145	H-3 → L	$d_{x^2-y^2} \to \pi^*$
			4	141 → 146	H-3 → L+1	$d_{x^2-y^2} \to d_{xy}$-n+π^*
T_8	2.54	488.3	16	141 → 145	H-3 → L	$d_{x^2-y^2} \to \pi^*$
			15	141 → 146	H-3 → L+1	$d_{x^2-y^2} \to d_{xy}$-n+π^*
			5	141 → 147	H-3 → L+2	$d_{x^2-y^2} \to d_{xy}$-n+π^*
			4	144 → 147	H → L+2	$d_{yz}+\pi \to d_{xy}$-n+π^*
T_9	2.61	475.6	15	143 → 146	H-1 → L+1	$\pi+d_{yz}/d_{z^2} \to d_{xy}$-n+$\pi^*$
			10	143 → 147	H-1 → L+2	$\pi+d_{yz}/d_{z^2} \to d_{xy}$-n+$\pi^*$
			9	143 → 148	H-1 → L+3	$\pi+d_{yz}/d_{z^2} \to \sigma^*(d_{z^2})$+ n
			6	142 → 147	H-2 → L+2	$d_{xz}+\pi \to d_{xy}$-n+π^*
T_{10}	2.71	457.6	21	142 → 148	H-2 → L+3	$d_{xz}+\pi \to \sigma^*(d_{z^2})$+ n
			13	142 → 147	H-2 → L+2	$d_{xz}+\pi \to d_{xy}$-n+π^*
			7	143 → 148	H-1 → L+3	$\pi+d_{yz}/d_{z^2} \to \sigma^*(d_{z^2})$+ n
T_{11}	2.73	453.5	18	143 → 146	H-1 → L+1	$\pi+d_{yz}/d_{z^2} \to d_{xy}$-n+$\pi^*$
			15	143 → 148	H-1 → L+3	$\pi+d_{yz}/d_{z^2} \to \sigma^*(d_{z^2})$+ n
T_{12}	2.77	448.2	19	144 → 147	H → L+2	$d_{yz}+\pi \to d_{xy}$-n+π^*
			13	144 → 148	H → L+3	$d_{yz}+\pi \to \sigma^*(d_{z^2})$+ n
			7	140 → 145	H-4 → L	$n_{Rib}/\sigma_{Rib} \to \pi^*$
T_{13}	2.83	438.6	32	140 → 145	H-4 → L	$n_{Rib}/\sigma_{Rib} \to \pi^*$
			6	143 → 147	H-1 → L+2	$\pi+d_{yz}/d_{z^2} \to d_{xy}$-n+$\pi^*$
			4	144 → 148	H → L+3	$d_{yz}+\pi \to \sigma^*(d_{z^2})$+ n
T_{14}	2.84	436.6	22	143 → 147	H-1 → L+2	$\pi+d_{yz}/d_{z^2} \to d_{xy}$-n+$\pi^*$
			9	140 → 145	H-4 → L	$n_{Rib}/\sigma_{Rib} \to \pi^*$
			7	143 → 148	H-1 → L+3	$\pi+d_{yz}/d_{z^2} \to \sigma^*(d_{z^2})$+ n
			4	142 → 148	H-2 → L+3	$d_{xz}+\pi \to \sigma^*(d_{z^2})$+ n
T_{15}	2.92	424.5	21	142 → 147	H-2 → L+2	$d_{xz}+\pi \to d_{xy}$-n+π^*
			16	142 → 148	H-2 → L+3	$d_{xz}+\pi \to \sigma^*(d_{z^2})$+ n
			4	142 → 146	H-2 → L+1	$d_{xz}+\pi \to d_{xy}$-n+π^*
			4	143 → 147	H-1 → L+2	$\pi+d_{yz}/d_{z^2} \to d_{xy}$-n+$\pi^*$
T_{16}	3.01	411.4	20	141 → 148	H-3 → L+3	$d_{x^2-y^2} \to \sigma^*(d_{z^2})$+ n
			12	141 → 147	H-3 → L+2	$d_{x^2-y^2} \to d_{xy}$-n+π^*
			6	138 → 145	H-6 → L	$\pi+ d_{yz} \to \pi^*$
T_{17}	3.03	408.6	26	138 → 145	H-6 → L	$\pi+ d_{yz} \to \pi^*$
			6	141 → 148	H-3 → L+3	$d_{x^2-y^2} \to \sigma^*(d_{z^2})$+ n
			6	139 → 145	H-5 → L	$\pi_{Im} \to \pi^*$

			4	141 → 147	H-3 → L+2	$d_{x^2-y^2} \rightarrow d_{xy}-n+\pi^*$
T_{18}	3.16	392.8	40	139 → 145	H-5 → L	$\pi_{Im} \rightarrow \pi^*$
			9	138 → 145	H-6 → L	$\pi+ d_{yz} \rightarrow \pi^*$
T_{19}	3.28	378.0	45	137 → 145	H-7 → L	$n_{Rib}/\sigma_{Rib}+d_{yz}/d_{z^2}+\pi \rightarrow \pi^*$
T_{20}	3.36	368.9	20	141 → 148	H-3 → L+3	$d_{x^2-y^2} \rightarrow \sigma^*(d_{z^2})+ n$
			20	141 → 147	H-3 → L+2	$d_{x^2-y^2} \rightarrow d_{xy}-n+\pi^*$
			7	141 → 146	H-3 → L+1	$d_{x^2-y^2} \rightarrow d_{xy}-n+\pi^*$
T_{21}	3.43	361.0	43	144 → 149	H → L+4	$d_{yz}+\pi \rightarrow \pi^*$
T_{22}	3.44	360.1	32	143 → 149	H-1 → L+4	$\pi+d_{yz}/d_{z^2} \rightarrow \pi^*$
			7	136 → 145	H-8 → L	$\pi+n_{Rib} \rightarrow \pi^*$
T_{23}	3.49	355.4	41	140 → 146	H-4 → L+1	$n_{Rib}/\sigma_{Rib} \rightarrow d_{xy}-n+\pi^*$
T_{24}	3.60	344.0	30	136 → 145	H-8 → L	$\pi+n_{Rib} \rightarrow \pi^*$
			12	143 → 149	H-1 → L+4	$\pi+d_{yz}/d_{z^2} \rightarrow \pi^*$
T_{25}	3.62	342.2	19	140 → 147	H-4 → L+2	$n_{Rib}/\sigma_{Rib} \rightarrow d_{xy}-n+\pi^*$
			19	140 → 148	H-4 → L+3	$n_{Rib}/\sigma_{Rib} \rightarrow \sigma^*(d_{z^2})+ n$
T_{26}	3.71	334.5	26	140 → 147	H-4 → L+2	$n_{Rib}/\sigma_{Rib} \rightarrow d_{xy}-n+\pi^*$
			17	140 → 148	H-4 → L+3	$n_{Rib}/\sigma_{Rib} \rightarrow \sigma^*(d_{z^2})+ n$
T_{27}	3.74	331.2	29	138 → 146	H-6 → L+1	$\pi+ d_{yz} \rightarrow d_{xy}-n+\pi^*$
			9	139 → 146	H-5 → L+1	$\pi_{Im} \rightarrow d_{xy}-n+\pi^*$
T_{28}	3.79	327.5	43	142 → 149	H-2 → L+4	$d_{xz}+\pi \rightarrow \pi^*$
T_{29}	3.84	322.5	36	139 → 146	H-5 → L+1	$\pi_{Im} \rightarrow d_{xy}-n+\pi^*$
			11	138 → 146	H-6 → L+1	$\pi+ d_{yz} \rightarrow d_{xy}-n+\pi^*$
T_{30}	3.87	320.0	34	135 → 145	H-9 → L	$\sigma(d_{z^2})+n_{Rib}+\pi \rightarrow \pi^*$
			6	138 → 147	H-6 → L+2	$\pi+ d_{yz} \rightarrow d_{xy}-n+\pi^*$
			4	136 → 145	H-8 → L	$\pi+n_{Rib} \rightarrow \pi^*$
T_{31}	3.92	316.1	32	137 → 146	H-7 → L+1	$n_{Rib}/\sigma_{Rib}+d_{yz}/d_{z^2}+\pi \rightarrow d_{xy}-n+\pi^*$
			5	140 → 148	H-4 → L+3	$n_{Rib}/\sigma_{Rib} \rightarrow \sigma^*(d_{z^2})+ n$
T_{32}	3.97	312.4	18	138 → 147	H-6 → L+2	$\pi+ d_{yz} \rightarrow d_{xy}-n+\pi^*$
			13	139 → 147	H-5 → L+2	$\pi_{Im} \rightarrow d_{xy}-n+\pi^*$
			8	135 → 145	H-9 → L	$\sigma(d_{z^2})+n_{Rib}+\pi \rightarrow \pi^*$
T_{33}	4.00	309.8	23	138 → 148	H-6 → L+3	$\pi+ d_{yz} \rightarrow \sigma^*(d_{z^2})+ n$
			6	137 → 146	H-7 → L+1	$n_{Rib}/\sigma_{Rib}+d_{yz}/d_{z^2}+\pi \rightarrow d_{xy}-n+\pi^*$
			6	139 → 147	H-5 → L+2	$\pi_{Im} \rightarrow d_{xy}-n+\pi^*$
			4	137 → 147	H-7 → L+2	$n_{Rib}/\sigma_{Rib}+d_{yz}/d_{z^2}+\pi \rightarrow d_{xy}-n+\pi^*$
T_{34}	4.01	309.3	5	137 → 146	H-7 → L+1	$n_{Rib}/\sigma_{Rib}+d_{yz}/d_{z^2}+\pi \rightarrow d_{xy}-n+\pi^*$
			9	137 → 148	H-7 → L+3	$n_{Rib}/\sigma_{Rib}+d_{yz}/d_{z^2}+\pi \rightarrow \sigma^*(d_{z^2})+ n$
			6	138 → 147	H-6 → L+2	$\pi+ d_{yz} \rightarrow d_{xy}-n+\pi^*$
			8	138 → 148	H-6 → L+3	$\pi+ d_{yz} \rightarrow \sigma^*(d_{z^2})+ n$
			12	139 → 148	H-5 → L+3	$\pi_{Im} \rightarrow \sigma^*(d_{z^2})+ n$
T_{35}	4.04	307.0	27	139 → 147	H-5 → L+2	$\pi_{Im} \rightarrow d_{xy}-n+\pi^*$
			11	138 → 147	H-6 → L+2	$\pi+ d_{yz} \rightarrow d_{xy}-n+\pi^*$
T_{36}	4.10	302.3	38	144 → 150	H → L+5	$d_{yz}+\pi \rightarrow \pi^*_{Im}$
			6	139 → 148	H-5 → L+3	$\pi_{Im} \rightarrow \sigma^*(d_{z^2})+ n$
T_{37}	4.11	301.9	16	141 → 149	H-3 → L+4	$d_{x^2-y^2} \rightarrow \pi^*$
			14	139 → 148	H-5 → L+3	$\pi_{Im} \rightarrow \sigma^*(d_{z^2})+ n$
			9	144 → 150	H → L+5	$d_{yz}+\pi \rightarrow \pi^*_{Im}$
			5	138 → 148	H-6 → L+3	$\pi+ d_{yz} \rightarrow \sigma^*(d_{z^2})+ n$
T_{38}	4.11	301.7	32	141 → 149	H-3 → L+4	$d_{x^2-y^2} \rightarrow \pi^*$
			10	139 → 148	H-5 → L+3	$\pi_{Im} \rightarrow \sigma^*(d_{z^2})+ n$
T_{39}	4.13	300.3	50	134 → 145	H-10 → L	$n_{Rib}/\sigma_{Rib} \rightarrow \pi^*$
T_{40}	4.16	298.3	40	143 → 150	H-1 → L+5	$\pi+d_{yz}/d_{z^2} \rightarrow \pi^*_{Im}$

Table S5. MO energy and fragments contributions for model structure of RibCbl calculated in gas phase employing the DFT/BP86/TZVP level of theory.

MOs		Orbital energy E [eV]	Character of MOs	Contributions of fragments in MO [%]				
				Co	C_{Rib}	Rib	Im	Corrin
134	HOMO-10	-9.24	π+ n_{Rib}/σ_{Rib}+d_{xz}/d_{z^2}	8	6	30	2	60
135	HOMO-9	-9.06	n_{Rib}/σ_{Rib}	2	2	78	1	20
136	HOMO-8	-8.97	$\sigma(d_{z2})$+n_{Rib}+π	12	9	69	4	14
137	HOMO-7	-8.68	π_{Im}	1	0	1	89	9
138	HOMO-6	-8.55	π+ d_{yz}	25	4	7	3	65
139	HOMO-5	-8.4	n_{Rib}/σ_{Rib}+d_{xz}/d_{z^2}+π	4	12	86	1	10
140	HOMO-4	-7.92	$d_{x^2-y^2}$	51	0	11	0	37
141	HOMO-3	-7.88	n_{Rib}/σ_{Rib}+ $d_{x^2-y^2}$+π	8	2	68	0	25
142	HOMO-2	-7.62	d_{xz}+π	43	3	10	1	45
143	HOMO-1	-7.45	π+d_{xz}/d_{z^2}	8	4	16	10	66
144	HOMO	-7.37	d_{yz}+π	36	2	11	5	48
145	LUMO	-5.37	π^*	4	1	1	3	92
146	LUMO+1	-4.65	d_{xy}-n+π^*	22	1	2	2	74
147	LUMO+2	-4.49	d_{xy}-n+π^*	18	1	2	2	78
148	LUMO+3	-4.37	$\sigma^*(d_{z2})$+ n	28	20	25	6	41
149	LUMO+4	-3.83	π^*	2	0	3	1	94
150	LUMO+5	-3.48	π^*_{Im}	0	0	0	74	26

Table S6. MO energy and fragments contributions for model structure of RibCbl calculated in water solution (PCM) employing the DFT/BP86/TZVP level of theory.

MOs		Orbital energy E [eV]	Character of MOs	Contributions of fragments in MO [%]				
				Co	C_{Rib}	Rib	Im	Corrin
134	HOMO-10	-7.10	n_{Rib}/σ_{Rib}	0	1	97	0	3
135	HOMO-9	-6.90	$\sigma(d_{z^2})+n_{Rib}+\pi$	8	11	64	4	24
136	HOMO-8	-6.72	$\pi+n_{Rib}$	4	3	43	3	50
137	HOMO-7	-6.28	$n_{Rib}/\sigma_{Rib}+d_{yz}/d_{z^2}+\pi$	10	18	71	5	14
138	HOMO-6	-6.17	$\pi+ d_{yz}$	22	3	17	7	55
139	HOMO-5	-6.12	π_{Im}	4	1	2	76	17
140	HOMO-4	-5.80	n_{Rib}/σ_{Rib}	4	1	74	0	22
141	HOMO-3	-5.54	$d_{x^2-y^2}$	65	1	4	1	30
142	HOMO-2	-5.26	$d_{xz}+\pi$	42	4	12	2	44
143	HOMO-1	-5.06	$\pi+d_{yz}/d_{z^2}$	9	5	11	10	70
144	HOMO	-4.99	$d_{yz}+\pi$	35	2	14	5	46
145	LUMO	-2.96	π^*	4	0	1	3	92
146	LUMO+1	-2.26	$d_{xy}-n+\pi^*$	23	1	2	2	73
147	LUMO+2	-2.08	$d_{xy}-n+\pi^*$	18	3	5	2	75
148	LUMO+3	-2.02	$\sigma^*(d_{z^2})+ n$	25	19	25	5	45
149	LUMO+4	-1.41	π^*	1	0	3	1	95
150	LUMO+5	-0.88	π^*_{Im}	0	0	1	72	27

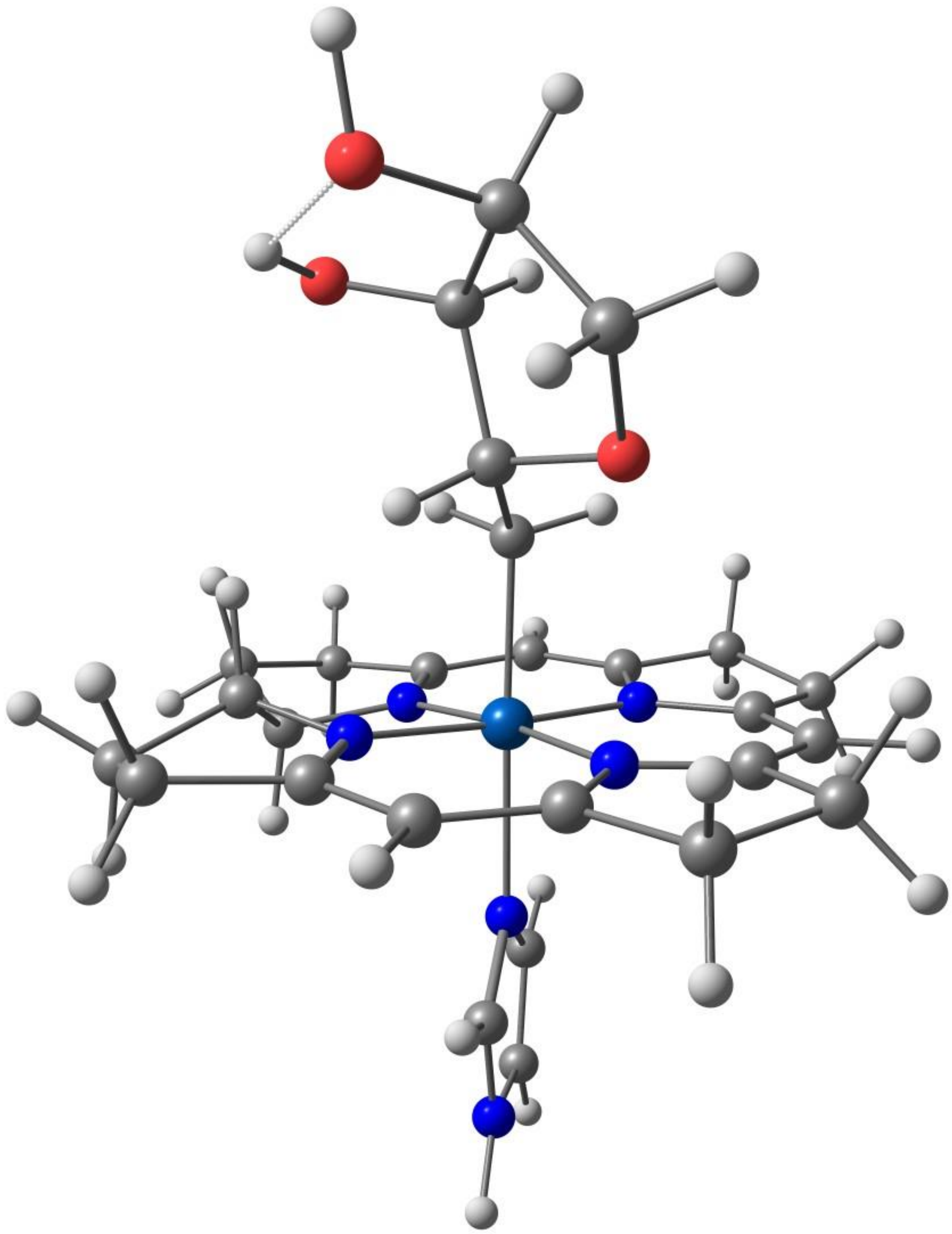

Figure S1. Molecular structure of RibCbl.

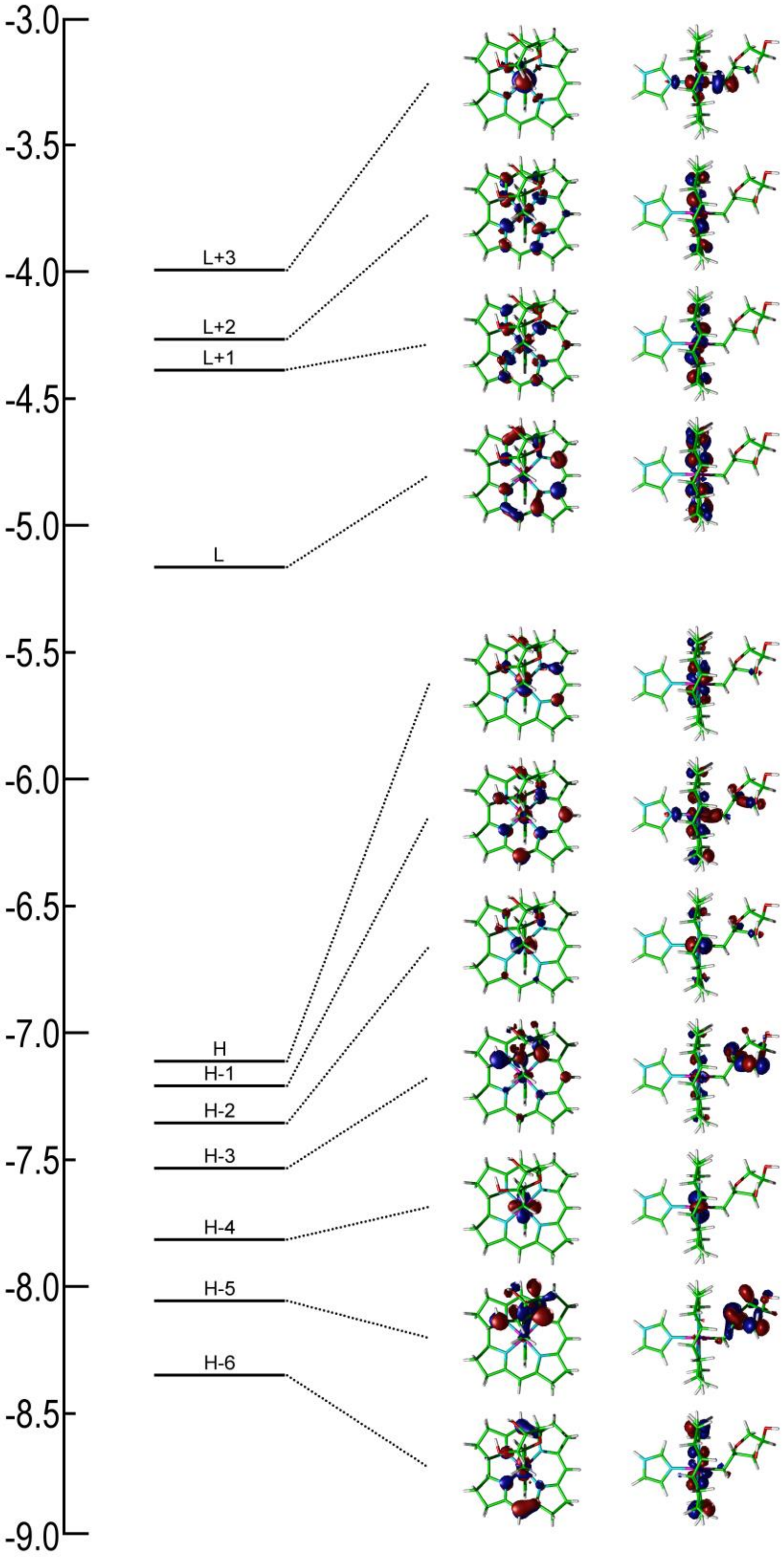

Figure S2. DFT–based isosurface plots of relevant MOs of gas phase Im-[Co^{III}(corrin)]-Rib^{+} obtained by DFT/BP86/TZVP level of theory. The MOLEKEL program was used to generate and visualize molecular orbitals.
